# IL-33 protects from recurrent *C*. *difficile* infection by restoration of humoral immunity

**DOI:** 10.1172/JCI184659

**Published:** 2025-03-06

**Authors:** Farha Naz, Md Jashim Uddin, Nicholas Hagspiel, Mary K. Young, David Tyus, Rachel Boone, Audrey C. Brown, Girija Ramakrishnan, Isaura Rigo, Claire Fleming, Gregory R. Madden, William A. Petri

**Affiliations:** 1 Department of Medicine, Division of Infectious Diseases and International Health,; 2 Department of Microbiology, Immunology, and Cancer Biology, and; 3 Department of Pathology, University of Virginia School of Medicine, Charlottesville, Virginia, USA.

**Keywords:** Immunology, Infectious disease, Adaptive immunity

## Abstract

*Clostridioides difficile* infection (CDI) recurs in 1 of 5 patients. Monoclonal antibodies targeting the virulence factor TcdB reduce disease recurrence, suggesting that an inadequate anti-TcdB response to CDI leads to recurrence. In patients with CDI, we discovered that IL-33 measured at diagnosis predicts future recurrence, leading us to test the role of IL-33 signaling in the induction of humoral immunity during CDI. Using a mouse recurrence model, IL-33 was demonstrated to be integral for anti-TcdB antibody production. IL-33 acted via ST2^+^ ILC2 cells, facilitating germinal center T follicular helper (GC-Tfh) cell generation of antibodies. IL-33 protection from reinfection was antibody-dependent, as μMT KO mice and mice treated with anti-CD20 mAb were not protected. These findings demonstrate the critical role of IL-33 in generating humoral immunity to prevent recurrent CDI.

## Abstract 2

Il-33 predicts recurrent CDI in patients, and the mice model showed that IL-33 promotes anti-TcdB antibody production via ILC2 and GC-Tfh cells, which is crucial for preventing reinfection.

## Graphical abstract

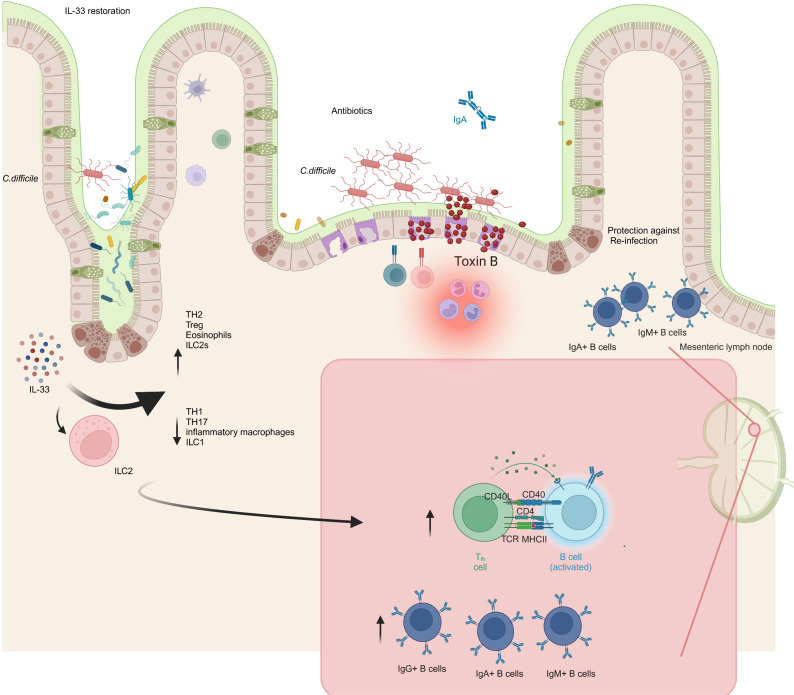

## Introduction

A unique and challenging aspect of *Clostridioides difficile* infection (CDI) is its tendency to recur in up to 25% of patients, a risk that increases with each subsequent recurrent episode (1, 2). Antibiotics targeting *C*. *difficile* are a double-edged sword in CDI, which, despite treating the acute infection, further disrupt the intestinal microbiome, predisposing patients to recurrence (3). The most effective therapy to prevent recurrent CDI (rCDI) is fecal microbiota transplantation (FMT) and newer microbiota therapeutics (e.g., SER109 and RBX2660); however, these are not 100% efficacious and have significant limitations (i.e., cost/logistical barriers and the risk of transmitting pathogens in the case of FMT) (4, 5).

Recurrent infection can partly be attributed to compromised adaptive immunity, suggesting a role for the immune system in preventing and managing repeated infections (6). *C*. *difficile* toxin B plays a key role in CDI pathogenesis (7–9), and exogenous IgG antibodies against toxin B are capable of averting rCDI (10, 11). Investigation into the role of immunoglobulins IgA, IgG, and IgM has been a consistent focus in studies involving patients with CDI (10). For example, reduced levels of antibodies (IgG, IgA) against TcdA and TcdB in the serum were linked to recurrence, whereas antibodies targeting cell surface antigens did not show any such correlation (12). Recent findings indicate that the presence of toxin B–specific IgG during acute CDI correlates with a delay in the onset of recurrence (11, 13).

Previous work has shown that IL-33 prevents mortality and epithelial disruption by activating group 2 innate lymphoid cells (ILC2s) in the acute mouse model of CDI (14). The microbiota influences IL-33 expression, and dysregulated IL-33 signaling predicts acute *C*. *difficile*–associated mortality in humans (14), emphasizing its crucial role in the defense against acute CDI. In this investigation, it is demonstrated that the type 2 alarmin IL-33 serves as a biomarker of recurrence in humans. Additionally, utilizing a mouse model, the pivotal role of IL-33 in antibody-dependent protection from recurrence is elucidated.

## Results

### IL-33–induced toxin-specific antibodies in the C. difficile mouse model.

Studies have shown that IL-33 triggers the activation of ILC2s (14), which have the potential to enhance humoral immunity (15). Antibodies to *C*. *difficile* toxin B are known to prevent recurrence (10). We hypothesized that IL-33 promotes ILC2-dependent antitoxin antibody production. In the mouse model of acute (primary) CDI, antibiotics induce susceptibility by decreasing IL-33 and subsequent IL-33 activation of ILC2 (14). The antibiotic-induced deficiency in IL-33 in acute CDI could therefore predispose to recurrent infection by impairing the production of antitoxin B antibody.

**Figure 1:**
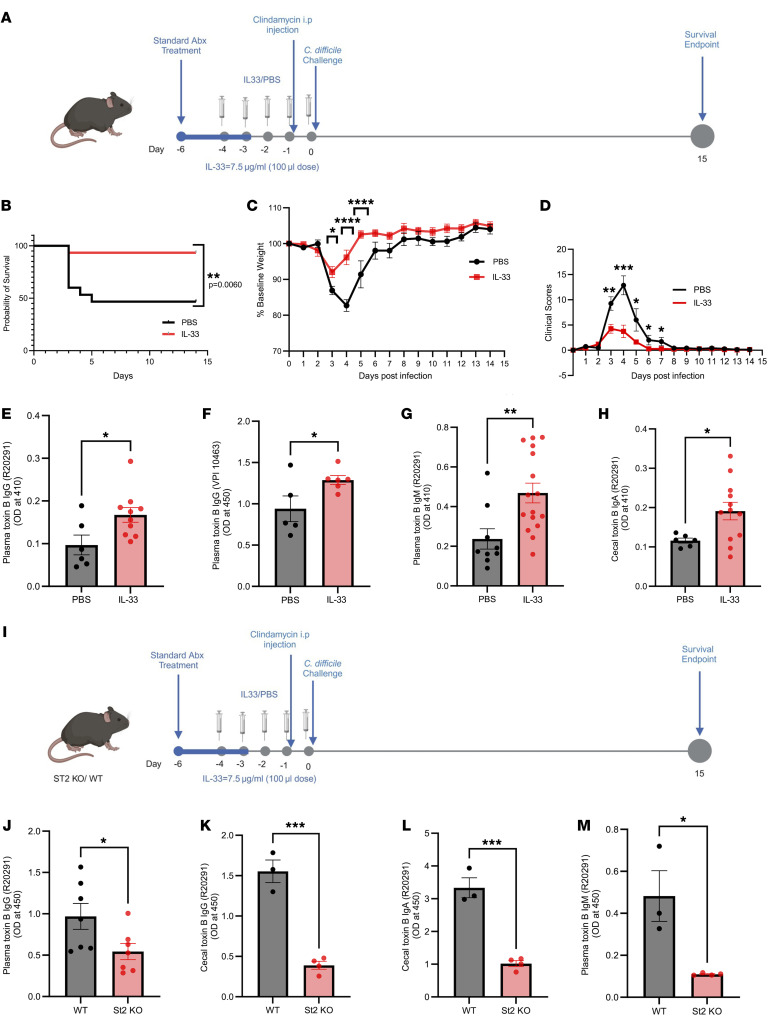
IL-33 increases toxin-specific antibody in mice after first infection with *C*. *difficile*. IL-33 (0.75 μg) was administered i.p. on days –4 to 0 to C57BL/6J mice (**A**–**H**) and/or ST2^−/−^ mice (**I–M**). Mice were infected with *C*. *difficile* strain R20291 (**A**–**E** and **G**–**M**) or VPI 10463 (**F**). On day 15 after infection, antibodies were measured in plasma and cecal content. (**A**) Schematic diagram showing infection and treatment timeline; (**B**) survival curves; (**C**) weight loss; (**D**) clinical scores; (**E**) plasma toxin B–specific IgG from mice infected with *C*. *difficile* strain R20291; (**F**) plasma toxin B–specific IgG from mice infected with *C*. *difficile* strain VPI 10463; (**G**) plasma toxin B–specific IgM from mice infected with *C*. *difficile* strain R20291; (**H**) cecal content toxin B–specific IgA from mice infected with *C*. *difficile* strain R20291. (**I**–**M**) WT versus ST2^−/−^ mice infected with *C*. *difficile* strain R20291: (**I**) Schematic diagram showing infection and treatment timeline; (**J**) plasma IgG; (**K**) cecal IgG; (**L**) cecal IgA; and (**M**) plasma IgM. (**B**) Comparison made by log-rank test (*n* = 30). (**C** and **D**) Comparison made by 2-tailed Student’s *t* test (**C** and **D** *n* = 30). **E** (*n* = 16), **F** (*n* = 11), **G** (*n* = 25), **H** (*n* = 18), **J** (*n* = 14), **K** (*n* = 7), **L** (*n* = 7), **M** (*n* = 7), A 2-tailed *t* test for normally distributed data and a Mann-Whitney test for nonnormally distributed data were used. *P < 0.05, **P < 0.01, and ***P < 0.001. The error bar indicates SEM.

To investigate the role of IL-33 in antitoxin B antibody production, IL-33 was first supplemented in the acute CDI mouse model. Prior to infection with the hypervirulent epidemic R20291 strain, mice were given antibiotics, and IL-33 protein was administered daily for 5 days by intraperitoneal injection (0.75 μg/mouse) (Figure 1A). IL-33 treatment reconstituted the antibiotic-depleted IL-33 protein level within the colon before infection (Supplemental Figure 1A; supplemental material available online with this article; https://doi.org/10.1172/JCI184659DS1). As previously observed, acute CDI was less severe in IL-33–treated mice as shown in the survival curve, weight loss, and clinical scores between the groups (Figure 1, B–D) (14). Toxin B–specific antibodies (IgG, IgM, and IgA) 15 days after infection were higher in cecal contents and plasma of IL-33–treated mice (Figure 1, E, G, and H). A similar IL-33 induction of antitoxin B antibody (IgG) was seen after infection with the classical *C*. *difficile* strain VPI 10463 (Figure 1F). Survival, weight loss, and clinical scores for VPI strain infection were found similar to those of R20291 (data not shown). *C*. *difficile* burden at day 15 after infection was unaltered by IL-33 (Supplemental Figure 1, B and C). It is concluded that the administration of IL-33 at the time of antibiotic pretreatment protected from acute CDI and enhanced the production of antitoxin antibodies.

The ST2 receptor for IL-33 is expressed on many immune cells including B cells (16). To test if the induction in antibody production was due to IL-33 signaling via its receptor ST2, we compared antibody production in ST2 knockout and WT mice following IL-33 administration (Figure 1I). Cecal contents and plasma were collected 15 days after infection. As expected, IL-33 did not induce the production of anti-TcdB antibodies (IgG, IgA, and IgM,) in ST2-KO mice (Figure 1, J–M). As a negative control, we infected ST2-KO mice without IL-33 treatment and observed no significant difference in Toxin B–specific antibody production (Supplemental Figure 1, D and E). This led to the conclusion that IL-33 exerts its effects through the ST2 receptor.

### Decrease in severity of C. difficile reinfection by IL-33.

**Figure 2:**
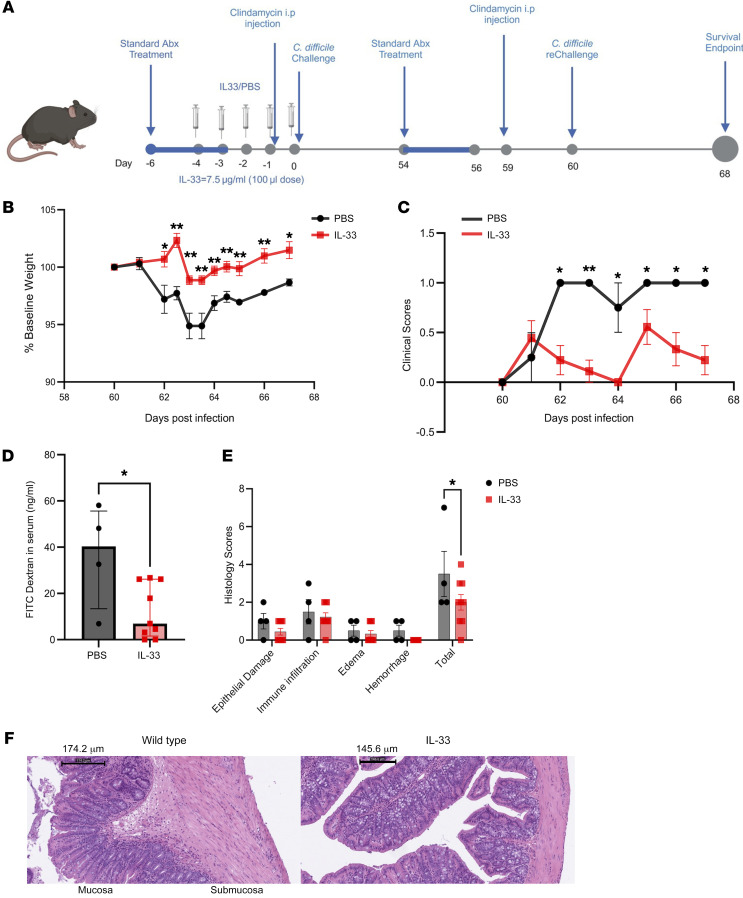
IL-33 protects from a second *C*. *difficile* infection. IL-33 (0.75 μg) was administered i.p. on days –4 to 0 and WT mice were infected on day 0 and again on day 60 with *C*. *difficile* strain R20291. (**A**) Experimental design for second infection; (**B**) second infection weight loss (*n* = 13); (**C**) clinical scores (*n* = 13); (**D**) FITC-dextran gut permeability test (*n* = 13); (**E**) epithelial damage scoring (*n* = 13); (**F**) representative H&E stain of the colon. Scale bars: 174.2 μm (left) and 145.6 μm (right). (**B** and **C**) Comparison made by 2-tailed Student’s *t* test. (**D**) Mann-Whitney test for nonnormally distributed data was used (**E**) Šídák’s multiple comparisons test was used to determine the statistical significance between groups. *P < 0.05, **P < 0.01, and ***P < 0.001. The error bar indicates SEM in **B**, **D**, and **E**, and in **D** indicates the median with interquartile range.

To test if restoration of IL-33 during acute CDI could protect from reinfection, a murine model of reinfection was utilized (Figure 2). C57BL/6J mice were infected on day 0 with *C*. *difficile* strain R20291 after pretreatment with antibiotics with or without exogenous IL-33 administration. First, we established the mouse reinfection model of *C*. *difficile*. Of note, we did not find any difference in bacterial colonization between the groups throughout the infection trajectory (data not shown). On day 54, after recovery from the primary infection, the mice were treated with antibiotics before reinfection with 1 × 10^4^ *C*. *difficile* spores from strain R20291 (Figure 2A). Antibiotic retreatment was important to clear the bacterial colonization, as *C*. *difficile* and toxin production were still detected even 100 days after primary infection using a bacterial quantification kit (TechLab Inc., TL5025) and an ELISA kit to detect toxin B in the stool (TechLab Inc., T5015). Unlike the control mice, mice treated with IL-33 during the primary acute CDI did not show clinical signs or lose weight upon reinfection (Figure 2, B and C). Further, IL-33 treatment during acute CDI led to improved gut barrier function during reinfection (Figure 2D). The treated group also experienced reduced submucosal edema and epithelial damage (Figure 2, E and F). It is concluded that IL-33 restoration during primary CDI promoted gut integrity to protect from reinfection.

We then tested if IL-33 could be used after a primary infection to prevent reinfection. IL-33 was administered after the acute infection prior to rechallenge (Supplemental Figure 2A). The group who received IL-33 before reinfection regained weight faster than the PBS recipient group (Supplemental Figure 2B) and returned to a clinical score of zero faster than the PBS group (Supplemental Figure 2C). The IL-33–treated group also produced more IgM and IgG in the serum than the control group (Supplemental Figure 2, D and E), suggesting that protection from reinfection may be achieved through increased toxin-specific antibody production. Of note, IL-33 did not alter the colonization of the bacteria.

### Importance of antibody production for IL-33 mediated protection against reinfection.

**Figure 3:**
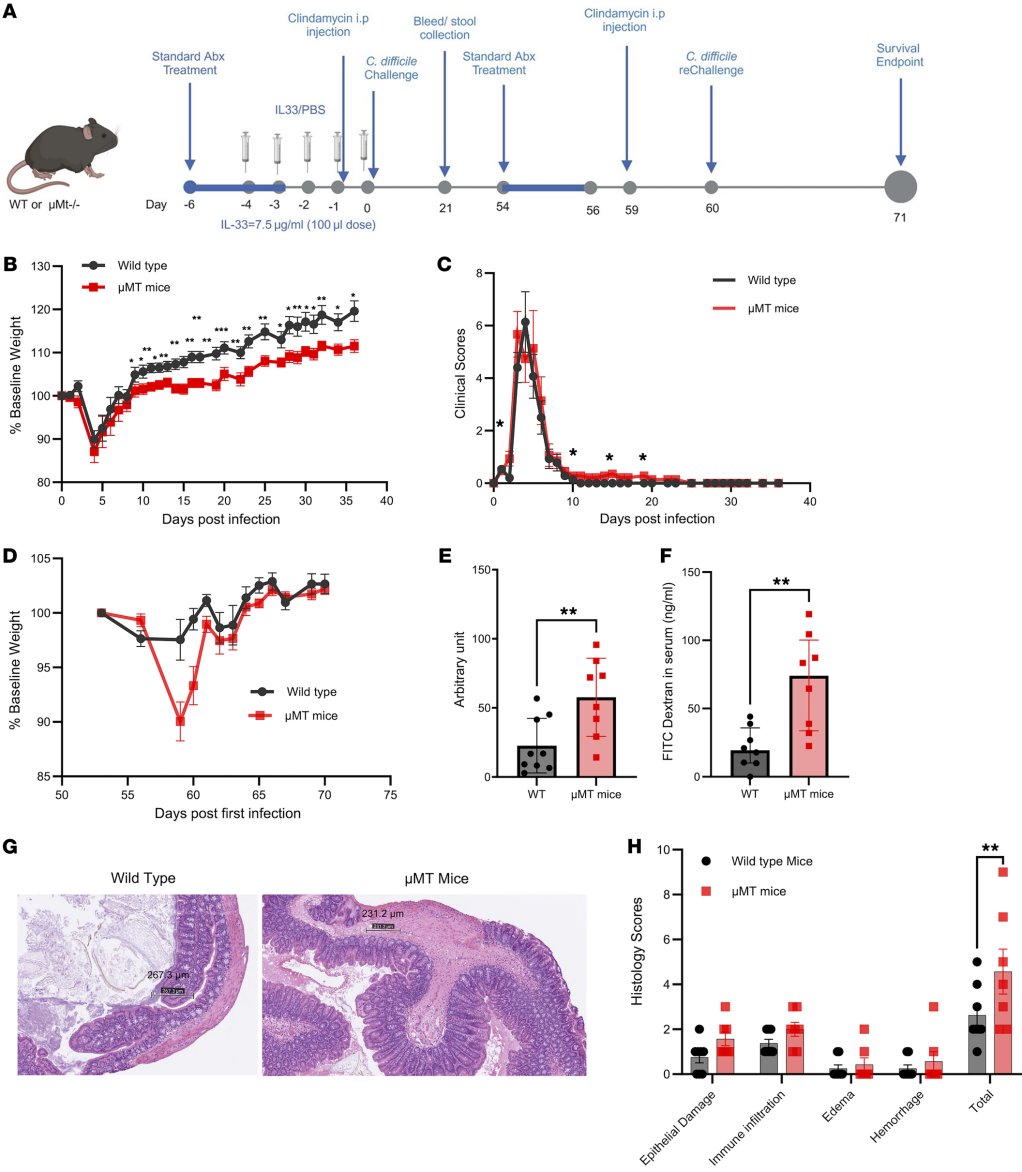
IL-33 protection from a second *C*. *difficile* infection is antibody dependent. WT (*n* = 15) and μMT-KO (*n* = 15) mice were administered IL-33 (0.75 μg) i.p. on days –4 to 0 and mice infected on day 0 and reinfected on day 60 with *C*. *difficile* strain R20291. (**A**) Experimental design; (**B**) first infection weight loss; (**C**) clinical scores. WT and μMT-KO mice were reinfected with *C*. *difficile* R20291 60 days after the first infection. Reinfection (**D**) weight loss; (**E**) Stool *C*. *difficile* toxin A and B measured by ELISA kit (Techlab); (**F**) FITC-dextran gut permeability assay; (**G**) H&E stain; (**H**) epithelial damage scoring. Scale bars: 267.3 μm (left) and 231.2 μm (right). (**B**–**D**) Comparison made by 2-tailed Student’s *t* test (**D** n=18). (**E**) a 2-tailed *t* test was used and the error bar indicates SEM. (**F**) a Mann-Whitney test was used and the error bar indicates the median with interquartile range. (**H**) Šídák’s multiple comparisons test was used. *P < 0.05, **P < 0.01, and ***P < 0.001.

In order to determine if IL-33–mediated protection against reinfection was mediated by antibody, μMT-KO mice that lack mature B cells were pretreated with IL-33 and infected with *C*. *difficile* (Figure 3A) During the first 10 days of the initial *C*. *difficile* infection, there was no difference in weight loss or clinical scores between WT and μMT-KO mice, consistent with prior work that showed no role of B cells and T cells in the acute phase of CDI (Figure 3, B and C) (14, 17). Interestingly, from day 11 onward, WT mice gained significantly more weight than the μMT-KO mice, suggesting a role of antibodies in the subacute recovery phase of primary CDI. To confirm the absence of antibodies, plasma and stool IgG, IgM, and IgA were measured from the μMT-KO mice that did not produce toxin B–specific antibodies (Supplemental Figure 3, A–E) (18). Mice were retreated with antibiotic cocktails and reinfected on day 60 after the primary *C*. *difficile* infection. The μMT-KO mice lost more weight than the WT mice when given antibiotics and reinfection (Figure 3D). Interestingly, μMT-KO mice had higher levels of toxin A/B in the stool (Figure 3E), had increased gut permeability calculated by FITC-dextran gut permeability assay (Figure 3F), and greater submucosal edema and epithelial damage (Figure 3, G and H). Samples were collected after the endpoint of the experiment, i.e., 11 days after reinfection. Surprisingly, μMT-KO mice had a lower *C*. *difficile* bacterial burden as measured by a GDH ELISA kit (TechLab Inc., TL502) (Supplemental Figure 3F).

**Figure 4:**
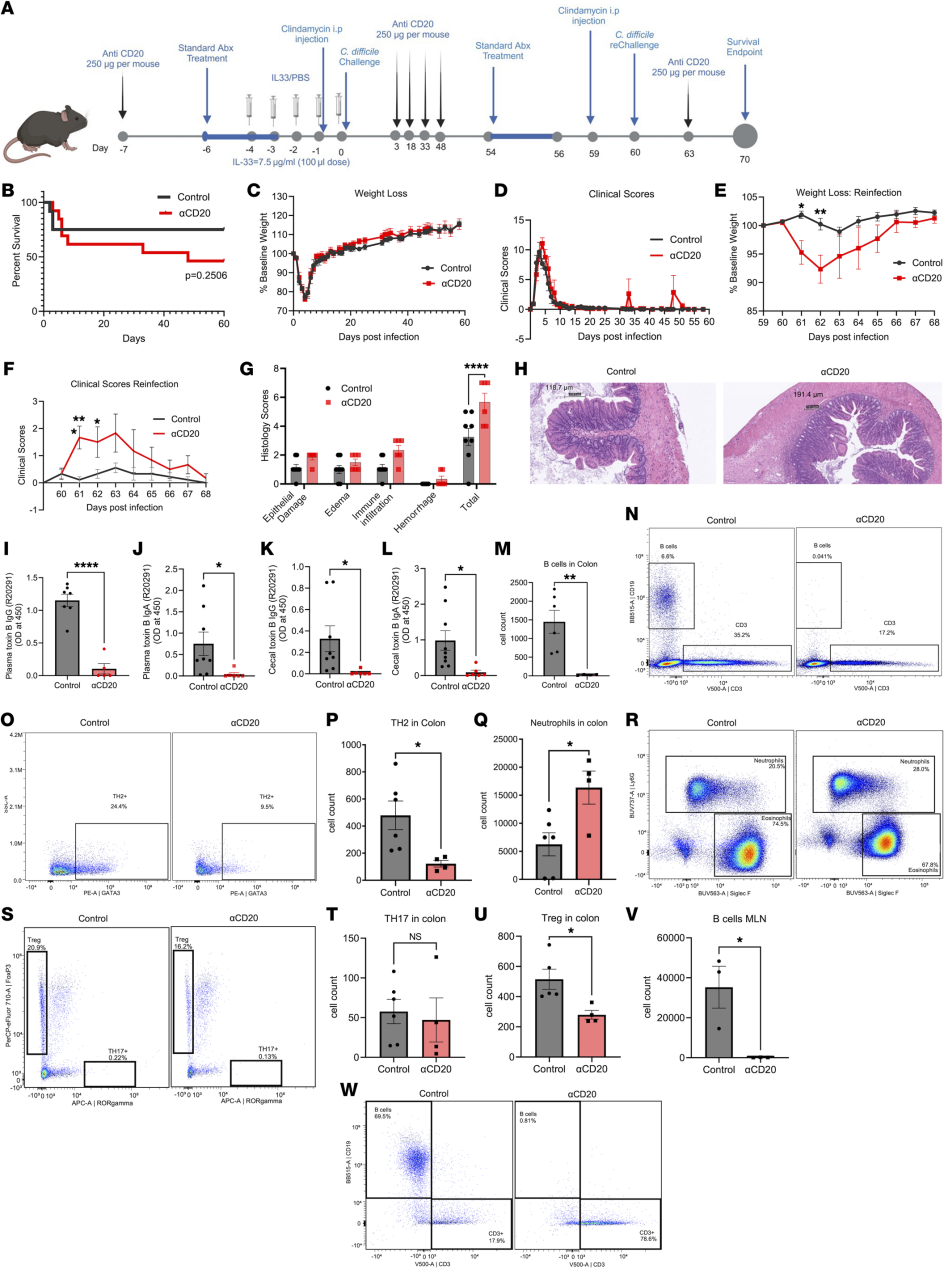
Antibody-deficient mice (anti-CD20 treated) lost IL-33–mediated protection from a second *C*. *difficile* infection. Anti-CD20 was administered to deplete B cells on days –7, 3, 18, 33, 48, and 63, and mice infected on day 0 and infected for the 2nd time on day 60 with *C*. *difficile* strain R20291. (**A**) Experimental design; (**B**) survival curve; (**C**) first infection weight loss; (**D**) first infection clinical scores; (**E**) reinfection weight loss; (**F**) reinfection clinical scores (**G**) epithelial damage scoring; (**H**) H&E stain. Scale bars: 118.7 μm (left) and 191.4 μm (right). (**I**) Toxin B–specific plasma IgG; (**J**) IgA; (**K**) cecal IgG; and (**L**) cecal IgA measured on day 10 after second infection. MLN and colon were harvested on day 10 after reinfection. Colonic (**M** and **N**) B cells (CD45^+^ CD3^–^ CD19^+^); (**O** and **P**) TH2 cells (CD45^+^CD3^+^ CD4^+^ GATA3^+^); (**Q** and **R**) neutrophils; (**S**–**U**) Treg (CD45^+^CD3^+^ CD4^+^ FOXP3^+^), and TH17 (CD45^+^CD3^+^ CD4^+^ RORγt^+^) cells; and (**V** and **W**) MLN B cells. (**B**) Comparison made by log-rank test (*n* = 26 in both groups). (**C**–**F**) Comparison made by 2-tailed Student’s t-test (**C** and **D** *n* = 26, **E** and **F** *n* = 15). (**G**) Šídák’s multiple comparisons test was used. (**I**–**M**, **P**, **Q**, **T**, **U**, and **V**) A 2-tailed *t* test for normally distributed data and a Mann-Whitney test for nonnormally distributed data were used. *P < 0.05, **P < 0.01, ***P < 0.001, and ****P < 0.0001. The error bar indicates SEM.

Given the limitations of μMT-KO mice in producing IgE and IgG antibodies (19), we employed anti-CD20 to deplete B cells (Figure 4A). During acute CDI, the anti-CD20–treated mice had slightly higher mortality (all but 1 of the mice were found dead) (Figure 4B), but no difference in weight loss and clinical scores was found (Figure 4, C and D). Upon reinfection, the anti-CD20–treated mice lost more weight (Figure 4E) and had higher clinical scores (Figure 4F) and worse submucosal edema and epithelial damage (Figure 4, G and H). No toxin-specific antibodies (IgG, IgA) were detected at the end of reinfection, as assessed from plasma and cecal tissue (Figure 4, I–L). B cell depletion was confirmed in the colon and MLN (Figure 4, M, N, V, and W). Interestingly, we observed that TH2 (Figure 4, O and P) and Treg cell populations (Figure 4, S and U) in the colon were significantly lower, and neutrophils were higher (Figure 4, Q and R) in the anti-CD20–treated mice, although no difference in TH17 cells was seen (Figure 4T). This finding indicated a greater degree of inflammation in the colon of anti-CD20–treated mice. The overall conclusion was drawn that antibody production was required for IL-33–mediated protection from recurrent *C*. *difficile*.

The next question was whether IL-33–mediated protection from recurrent *C*. *difficile* was strain specific. The hypervirulent strain R20291 produces toxin B (TcdB2), which is antigenically distinct from the TcdB1 produced by the classical strain VPI 10463. The expectation was that if IL-33 protection from recurrent R20291 infection was due to anti-TcdB2 antibody production, IL-33 would not prevent recurrence from the classical VPI 10463 that produces TcdB1. Forty days after a primary infection with *C*. *difficile* strain R20291 with IL-33 treatment, mice were reinfected with either R20291 or VPI 10463 (Supplemental Figure 4A). Mice reinfected with a different strain (i.e., VPI 10463) than the strain used in a primary infection (i.e. R20291) lost more weight (Supplemental Figure 4B) and had a more severe clinical score (Supplemental Figure 4C) than the group that was reinfected with the same strain (i.e., R20291). It is concluded that the strain specificity of IL-33–mediated protection against reinfection was consistent with its mediation by strain-specific antitoxin B antibodies.

### IL-33–mediated increase in mucosal type 2 immunity during primary C. difficile infection.

**Figure 5:**
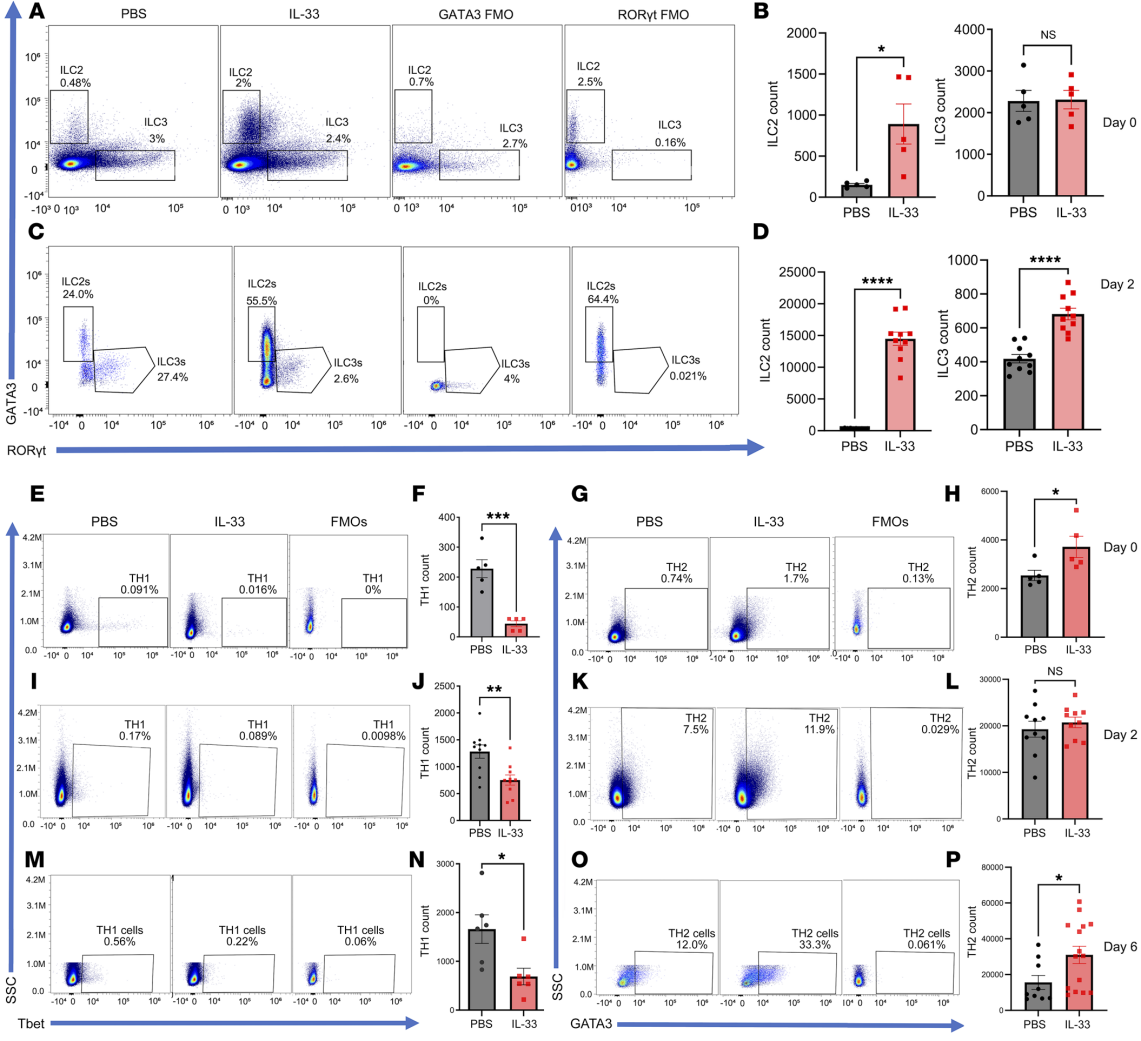
IL-33 increased mesenteric lymph node ILC2 and decreased TH1 cells during first *C*. *difficile* infection. IL-33 (0.75 μg) was administered i.p. on days –4 to 0 and mice infected on day 0. (**A** and **B**) ILC populations on day 0, prior to infection, and (**C** and **D**) at 2 daysafter infection. TH1, TH2 populations (**E**–**H**) before infection, day 0; (**I**–**L**) day 2; and (**M**–**P**) day 6 after infection. A 2-tailed *t* test for normally distributed data and a Mann-Whitney test (**B**, **F**, and **H** *n* = 10, **D**, **J**, and **L** *n* = 20) for nonnormally distributed data were used. *P < 0.05, **P < 0.01, ***P < 0.001, and ****P < 0.0001. The error bar indicates SEM.

**Figure 6:**
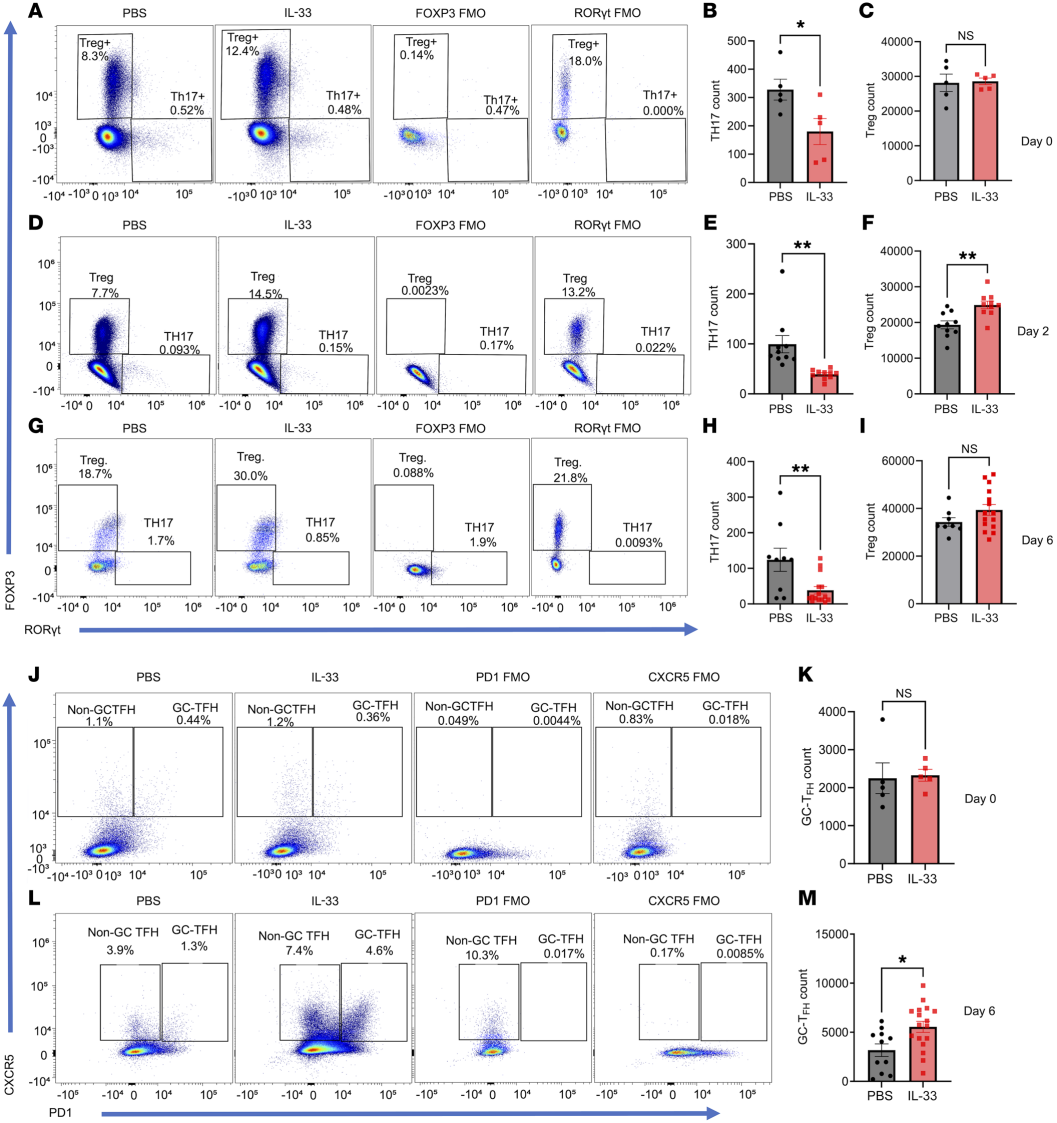
IL-33 increased mesenteric lymph node GC-TFH, Tregs, and decreased TH17 cells during a primary *C*. *difficile* infection. IL-33 (0.75 μg) was administered i.p. on days –4 to 0 and mice infected on day 0. Mesenteric lymph nodes were harvested to analyze T helper cells before infection, and again on day 2 and day 6 after first infection. (**A**–**C**) TH17 cells (CD45^+^ CD3^+^ CD4^+^ RORgt^+^) and Treg cells (CD45^+^ CD3^+^ CD4^+^ FOXP3^+^) on day 0 prior before infection (*n* = 10); (**D**–**F**) on day 2 (*n* = 20); (**G**–**I**) on day 6 after infection (*n* = 24). (**J**–**M**) TFH cells were defined as germinal center (GC) TFH (CD45^+^ CD3^+^ CD4^+^ CD44^+^ PD1 high CXCR5^+^) and non-GC TFH cells (CD45^+^ CD3^+^ CD4^+^ CD44^+^ PD1-low CXCR5^+^) by flow cytometry. (**J** and **K**) TFH subsets on day 0 prior to infection (*n* = 10); (**L** and **M**) TFH subsets on day 6 after infection(*n* = 28). A 2-tailed *t* test for normally distributed data and a Mann-Whitney test for nonnormally distributed data were used. *P < 0.05, **P < 0.01, and ***P < 0.001. The error bar indicates SEM.

To further understand cellular dynamics and the overall host response to the IL-33 treatment in dysbiotic mice before and after the first *C*. *difficile* challenge, immunophenotyping was done on the host’s innate and adaptive immune response. First, the immune population in infected and noninfected groups was checked after the recovery phase (16 days after infection). Neutrophils and TH17 cells were significantly higher in the *C*. *difficile–*infected mice even 16 days after infection, indicating that type 3 immunity dominates and persists beyond the resolution of CDI (Supplemental Figure 5, A–D). During primary CDI, IL-33 treatment led to an increase of ILC2s and a decrease in ILC1 and ILC3 populations in the mesenteric lymph nodes (MLN) (Figure 5, A–D) and colon (Supplemental Figure 6, A–D). Additionally, IL-33 remediation of dysbiosis led to an increase in TH2 and a decrease in TH1 cell populations in the MLN (Figure 5, E–P) and the colon (Supplemental Figure 6, E and F) both before and after the *C*. *difficile* challenge. Flow cytometry at days 2 and 6 after primary CDI demonstrated an IL-33–induced downregulation of TH17 cells and upregulation of Treg cells in MLN (Figure 6, A–I) and colon (Supplemental Figure 6, G–I). In line with our previous study, IL-33 increased colonic eosinophils and decreased inflammatory monocytes (Ly6C-high populations) (Supplemental Figure 6, J–M) (14). We concluded that IL-33 treatment during acute CDI promoted a long-lasting innate and adaptive type 2 immune response in the intestine and MLN.

### IL-33–mediated increase in activated mesenteric lymph node GC-TFH cells during primary C. difficile infection.

CDI induces an inferior IgG response and is associated with a lack of T follicular helper cell (TFH) expansion (20). We hypothesized that IL-33 protection from rCDI was due to TFH expansion to promote antitoxin B antibody. We chose to measure Tfh cells on days 0 and 6, as this is consistent with the expected time it takes for Tfh cells to differentiate in the germinal center (21, 22). In support of this hypothesis, we found that without IL-33 treatment, GC-TFH was 1.3% and with IL-33 treatment, GC-TFH was 4.6% of total activated GC-TFH (Figure 6, J–M). Gating strategies are described in Supplemental Figure 7.

Because IL-33 could activate dendritic cells via the ST2 receptor, we tested activation markers on dendritic cells before (day 0) and at 2, and 5 days of infection (Supplemental Figure 8). There was a significant influx of CD11c^+^ dendritic cells in MLN of the IL-33–treated group prior to the infection; however, only a small number of these dendritic cells expressed activation markers such as CD86 (Supplemental Figure 8, A–C). At day 2 after infection, significantly more CD11c^+^ dendritic cells with higher expressing activation marker CD86 were found in MLN (Supplemental Figure 8, D–F), but this difference was no longer observed by day 5 of infection (Supplemental Figure 8, G–I). We concluded that IL-33 promoted anti-toxin B antibodies by TFH expansion and in part also by recruiting dendritic cells to the MLN.

### Role of ILC2s in IL-33–mediated protection from reinfection.

We wanted to determine which primary or upstream cells responded to IL-33 via the ST2 receptor. We hypothesized that ILC2s respond to IL-33 to promote anti-toxin B antibodies and protect from reinfection, in part due to their known role in adaptive immunity (23) and due to the observed increase in ILC2s in the IL-33–treated group after infection in the MLN and colon (Figure 5, A–D, and Supplemental Figure 6, A and B).

**Figure 7:**
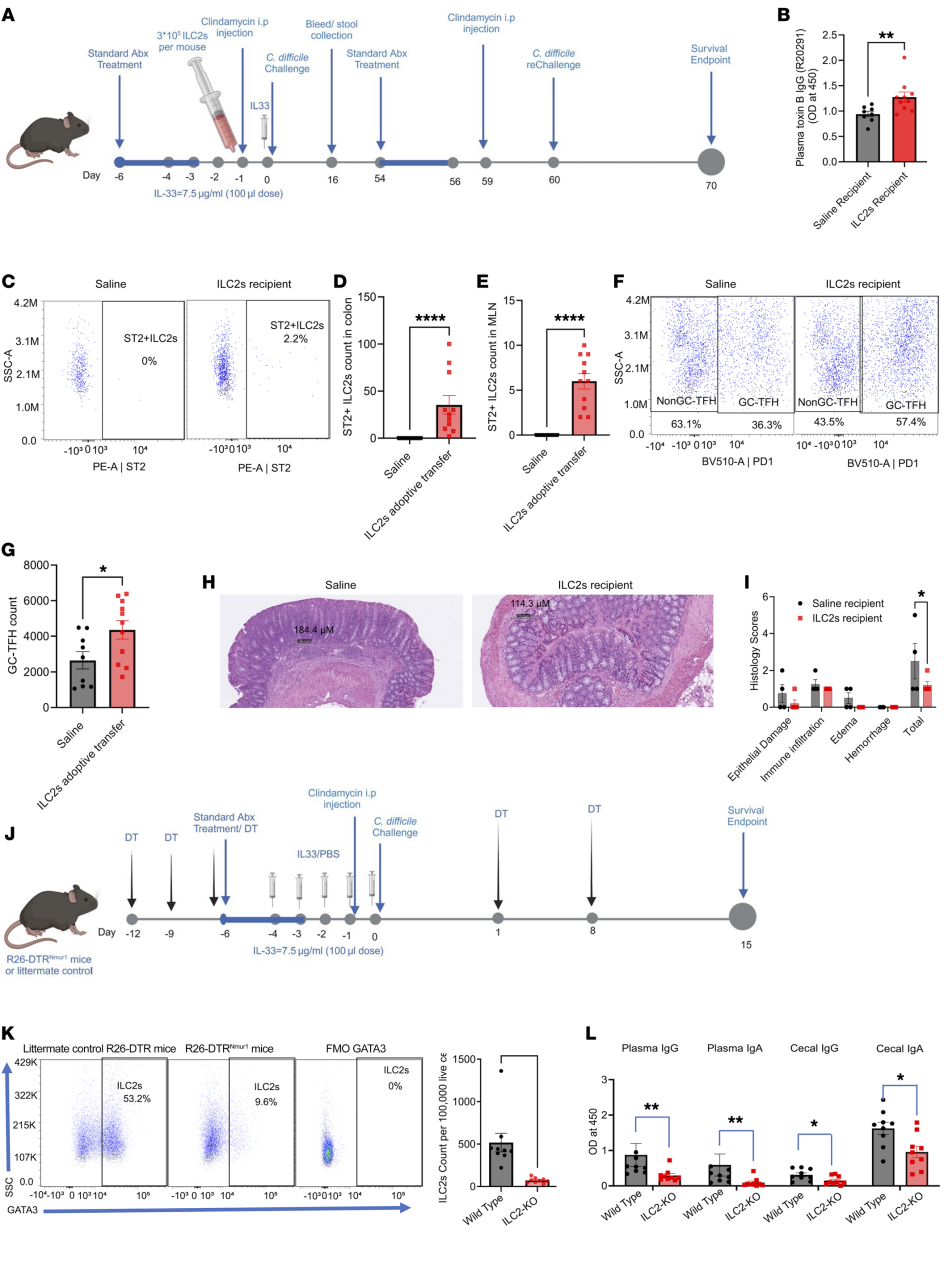
ILC2s mediated production of toxin B–specific antibodies, protecting against CDI reinfection. ST2^+^ ILC2s (from uninfected IL-33 treated mice) were ex-vivo expanded, purified by flow-sorting, and adoptively transferred into ST2-KO mice. (**A** and **B**) Mice were pretreated with antibiotics and injected with 0.75 μg per dose per mouse of IL-33 in the gut 1 day after the adoptive transfer of 3 × 10^5^ ILC2s (*n* = 12) or saline (*n* = 12) per mouse. At day 16 after primary infection, plasma toxin B–specific (**B**) IgG was measured in plasma. (**C**–**G**) Mice were rechallenged with *C*. *difficile* on day 60. On day 70 (10 days after the second infection), (**C**–**E**), ILC2 was measured in the colon and MLN. (**F** and **G**) GC-TFH measured in the MLN (**H**) Day 70 representative epithelial damage (H&E) of treatment groups and (**I**) assessed by blinded scoring of infected tissue. Scale bars: 184.4 μm (left) and 114.3 μm (right). Depletion of ILC2 decreased toxin-specific antibodies in CDI; IL-33 (0.75 μg) was administered intraperitoneally from days –4 to 0 to R26-DTR^Nmur1^ mice (*n* = 9) or littermate control R26-DTR mice (*n* = 9) (in which ILC2s lack DTR). Mice were then given intraperitoneal (i.p.) injections with diphtheria toxin (DT) on days –12, –9, –6, 1, and 8 after infection and infection was done with *C*. *difficile* strain R20291. (**J**) Experimental design; On day 15 after infection, ILC2 abundance and anti-TcdB antibodies were measured. (**K**) Density plot to show the depletion of ILC2s from the colon (**L**) Toxin-specific antibodies were measured in plasma and cecal content. A 2-tailed *t* test for normally distributed data and a Mann-Whitney test for nonnormally distributed data were used. (**J**) Šídák’s multiple comparisons test was used. *P < 0.05, **P < 0.01, and ***P < 0.001. The error bar indicates SEM.

To test the role of ILC2 in IL-33–mediated anti-toxin B antibody production, ST2^+^ ILC2s were isolated from the spleen, MLN, and colon of IL-33–treated mice, expanded in vitro (24, 25), flow sort purified (Supplemental Figure 9), and adoptively transferred into ST2^–/–^ mice (Figure 7A). Mice were infected with R20291 strain of *C. difficile* after a day of adoptive transfer of ST2^+^ ILC2s. During acute and reinfection, the ST2^+^ ILC2-recipient group had slightly less mortality (Supplemental Figure 10, A and B) but no difference in weight loss or clinical scores was found during the acute infection (Supplemental Figure 10, C and E). Whereas, upon reinfection, the ST2^+^ ILC2-recipient group showed a modest effect on weight (Supplemental Figure 10D), and clinical scores (Supplemental Figure 10F). ST2^–/–^ mice that received ST2^+^ ILC2s had increased plasma anti-toxin B IgG following primary challenge with *C*. *difficile* (Figure 7B). The presence of donor ST2^+^ ILC2s within the colon and MLN of recipient ST2^–/–^ mice was confirmed (Figure 7, C–E). ST2^+^ ILC2 recipient mice had an increase in activated GC-TFH population (Figure 7, F and G). Upon reinfection, ST2^+^ ILC2 recipient mice had improved gut permeability (Supplemental Figure 10G) and significantly less epithelial damage (Figure 7, H and I). We did not find any difference in the bacterial load and toxin level in the cecal content (Supplemental Figure 10, H and I).

To further validate the role of ILC2s in IL-33–mediated anti-toxin B antibody production, we utilized ROSA26-DTR^Nmur1^ mice (26). These mice expressed improved Cre (iCre) recombinase and enhanced GFP (eGFP) from the regulatory elements of Nmur1, allowing for selective Cre and reporter expression in ILC2s. The mice also have a loxP-flanked STOP cassette upstream of the open reading frame of the simian diphtheria toxin receptor (DTR) gene. Therefore, ILC2s selectively and constitutively express DTR, allowing for near-complete depletion of ILC2 upon administration of DT. Employing repeated injection of DT in Nmur1^iCre–eGFP^ROSA26^LSL–DTR^ (*n* = 10) mice or ROSA26^LSL–DTR^ littermates (*n* = 10) with IL-33 treatment and *C*. *difficile* infection, as shown in (Figure 7J), we validated depletion of ILC2s at the endpoint of the experiment (Figure 7K). We observed that depletion of ILC2s abrogated IL-33–induced toxin B specific antibody production estimated in plasma and cecal content antibodies collected at 15 days after infection. (Figure 7L). ILC2-depleted mice exhibit a limited impact on mortality (Supplemental Figure 10J) but increased morbidity based on weight loss (Supplemental Figure 10K) and clinical scores (Supplemental Figure 10L) measured during the acute infection. ILC2 depletion had no impact on the bacterial count (Supplemental Figure 10M) or toxin A/B level (Supplemental Figure 10N).

Both experiments conclusively demonstrate that IL-33 facilitates the production of toxin-specific antibodies through the activation of ILC2s. Moreover, findings from adoptive transfer experiments establish that these ILC2-mediated antibodies confer protective immunity against reinfection (Graphical abstract).

### IL-33 is a biomarker for recurrent C. difficile infection.

**Figure 8:**
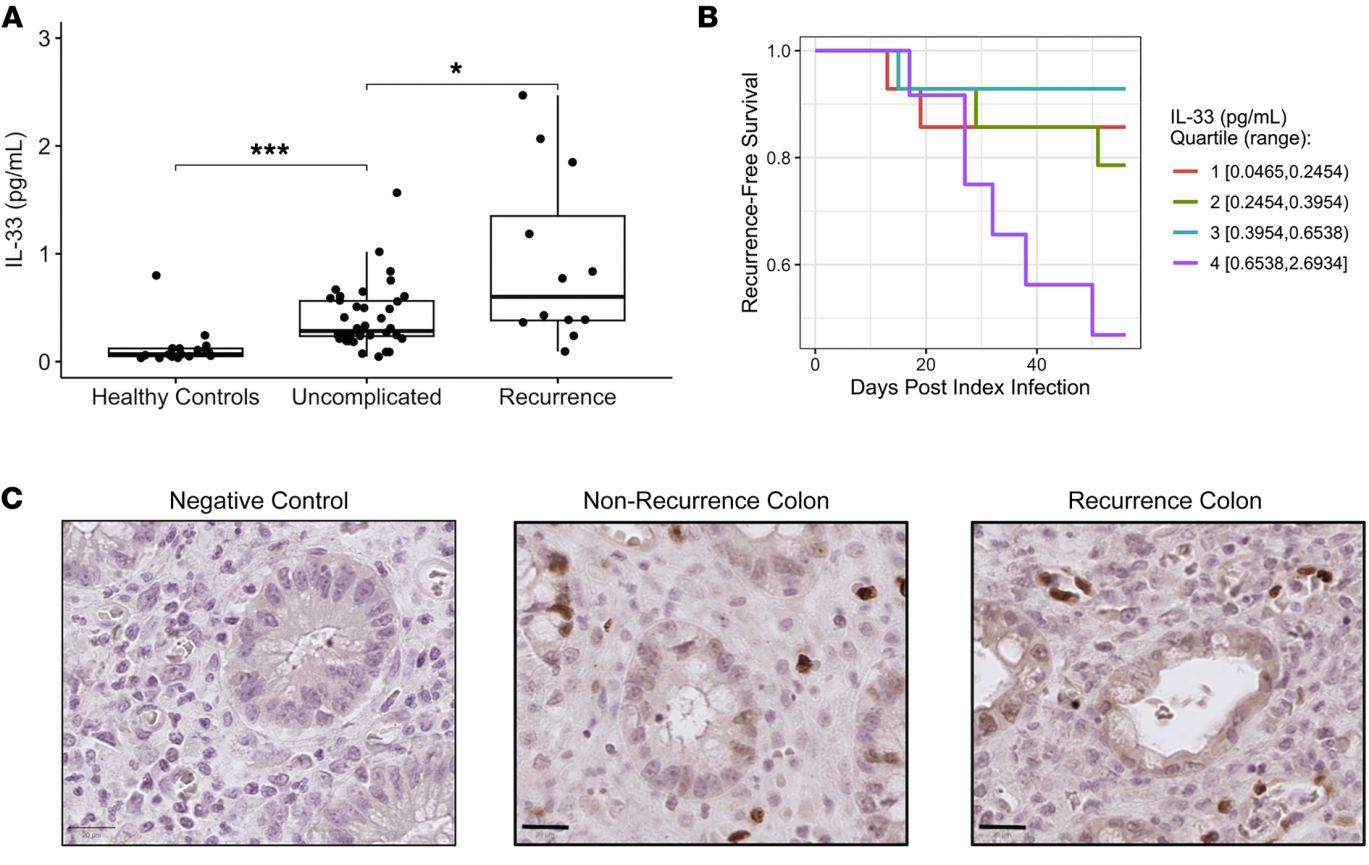
IL-33 is a biomarker for recurrent *C*. *difficile* infection in humans. (**A**) Plasma IL-33 was measured in healthy controls (*n* = 17), patients with uncomplicated CDI (*n* = 39), and recurrent CDI (*n* = 12) (excluding 5 patients who died) within 8 weeks of diagnosis. (**B**) Recurrence-free survival among the patients with C. difficile infection (1A), grouped by serum IL-33 quartile (Wilcoxon *P* = 0.002). (**C**) IHC staining of IL-33 from colon tissue biopsies of patients without or with recurrence. Scale bars: 20 μm. The Kaplan–Meier method was used to measure recurrence-free survival curves and to evaluate the effects of IL-33 on risk for recurrent *C*. *difficile* infection. To account for competing risk against recurrent infection, death within the 8-week follow-up period was treated as a censoring event.*P < 0.05 and ***P < 0.001.

Utilizing a commercial multiplex proximity extension assay, IL-33 was measured in the blood of 56 hospitalized patients with CDI (within 48 hours of diagnosis) and 17 people in a healthy control group. Patient details including demographics, comorbidities, and other information. are described in Supplemental Table 1. Among the *C*. *difficile*–infected patients, 12 developed recurrent infections, and 5 died within 8 weeks. IL-33 was elevated in uncomplicated CDI (median 0.309 pg/mL) compared with healthy controls (median 0.068 pg/mL; Wilcoxon *P* < 0.001). Only 3 out of the 45 cytokines measured were significantly different between patients who developed recurrent infection and those who did not. These cytokines included IL-33, C-X-C motif chemokine 10 (CXCL-10), C-C Motif Chemokine Ligand 3 (CCL3), and Tumor Necrosis Factor (TNF) (Supplemental Figure 11A). Supplemental Figure 11B presents ROC curve analysis for univariate and multivariable logistic regression models predicting recurrent *C*. *difficile* infection within 8 weeks. The AUC value for IL-33 was 0.72 (95% Cl: 0.53–0.9) and for IL-33 with CXCL10, CCL3, and TNF was 0.71 (95% Cl: 0.5–0.91). The univariate model, which includes only IL-33, performs similarly to the multivariable model that incorporates all 4 significant cytokines (IL-33, CXCL-10, CCL3, and TNF). This suggests that while CXCL-10, CCL3, and TNF are altered in patients with recurrent infection, they do not significantly improve the predictive power of the IL-33 model, which performs comparably in univariate and multivariable settings. IL-33 was higher in patients who went on to develop recurrent infection (median 0.600 pg/mL; *P* = 0.031) compared with uncomplicated/nonrecurrent infection (Figure 8, A and B). IHC staining revealed abundant anti–IL-33 staining of colonic epithelium from 3 nonrecurrent and 3 recurrent patients with CDI (Figure 8C). Different T cell populations, including Th1, Th2, and Th17 cells, were measured in peripheral blood samples from patients with nonrecurrent (uncomplicated CDI [*n* = 16], rCDI [*n* = 10], and controls [*n* = 15] (Supplemental Figure 12, A and B)). While the numbers of Th1, Th2, and Th17 cells were significantly lower in infected patients compared with controls, no significant differences were observed between patients with nonrecurrent or rCDI. Additionally, the percentage of CD4^+^ cells within these populations showed no significant variation across the groups. Based on these findings, we conclude that IL-33 serves as a biomarker for rCDI.

## Discussion

*C. difficile* patients with the highest quartile serum IL-33 levels (> 0.641 pg/mL) measured at diagnosis were more than 2.5 times more likely to develop recurrent infection within the following 8 weeks compared with patients with lower IL-33, suggesting that IL-33 could serve as an early biomarker for reinfection. The addition of CXCL-10 and TNF does not significantly improve the performance of a univariate IL-33 predictive model for recurrence. We employed a mouse model of reinfection to elucidate the role of IL-33 in rCDI. Our investigation revealed that IL-33 exerts a protective effect against CDI reinfection by activating ILC2s. ILC2 activation promoted humoral immunity against *C*. *difficile*. The transfer of ST2^+^ ILC2s to ST2^–/–^ mice alone stimulated the production of toxin B–specific antibodies and promoted the formation of T follicular helper (TFH) cell populations, which is consistent with earlier studies that ILC2 contributed to the development of T follicular helper (TFH) cells and antibody production (27).

The IL-33 signaling pathway plays a crucial role in promoting the humoral immune response and protecting against infection and reinfection by *C*. *difficile* colitis via the action of ILC2s. We observed that, after antibiotic dysbiosis, the administration of IL-33 resulted in elevated toxin B–specific antibody production, thereby conferring protection against toxin-induced epithelial damage and morbidity upon reinfection. To check whether the protection during reinfection is due to the IL-33–induced anti-toxin B antibodies, we utilized the μMT-KO mouse model where B cells were depleted. Considering the limitations of this model, as these mice could produce IgG and IgE antibodies (19), the anti-CD20-treatment mouse model was used to reconfirm the results by depleting *C*. *difficile*–specific B cells. An important note is that anti-CD20 does not deplete terminally differentiated antibody-producing B220^+^ plasmablast and plasma cells that do not express CD20 (28). These plasma cells could confer nonspecific B cell antibody–mediated protection. Combining both results, it was shown that IL-33–mediated protection during reinfection was due to toxin-specific antibodies. Our investigation corroborates earlier work showing that IL-33 facilitates the generation of IgA, contributing to the preservation of gut microbial homeostasis while mitigating IL-1α–induced colitis and colitis-associated cancer (29).

A likely target of humoral immunity is TcdB, which is recognized as the principal virulence determinant of *C*. *difficile*. TcdB has undergone expedited evolutionary changes, presumably in part to evade antibody neutralization. Clade 2 hypervirulent strains such as R20291 produce TcdB2, while VPI 10463 strains produce TcdB1 (2). The disparities in the sequences of the 2 TcdB forms result in modifications to antigenic epitopes, diminishing cross-neutralization effectiveness by antibodies directed toward the C-terminal domain (30). Consistent with IL-33 acting to prevent recurrence by promotion of anti-TcdB antibodies, IL-33 administration did not provide cross protection from a different TcdB type.

The interaction between antigen-stimulated B cells and the TFH cell subset determines the subsequent course of immune activity, including antibody production. TFH cells play a pivotal role in supporting activated B cells through cognate interactions, which involve antigen-specific binding, and the secretion of functionally significant cytokines, thereby coordinating and enhancing the humoral immune response (20). GC-TFH cells orchestrate B cells in germinal centers, facilitating somatic hypermutation and class-switch recombination, resulting in high-affinity antibodies. GC-TFH cells express specific molecules including CXCR5 and PD1, which enable their precise modulation of B cell interactions. Cytokines released by TFH augment B cell differentiation into plasma cells, ultimately amplifying antibody secretion (31). Research has also shown that IL-33 has the potential to boost humoral immunity through its interactions with TFH cells (32). We found an IL-33–dependent increase in antibody production through increased GC-TFH activity.

There are several important limitations to this study. IL-33 was upregulated in patients at the highest risk for rCDI, suggesting that IL-33 could predispose to recurrent infection. In addition, hospitalized patients with *C*. *difficile* infection were compared with people in a healthy control group but were not adjusted for age, comorbidities, and other factors. The absence of a non-*C*. *difficile* hospitalized patient comparator group limits our ability to contextualize the role of IL-33 outside of *C*. *difficile* infection; it remains unclear whether patient IL-33 levels were specific to this infection or if similar or higher levels may be present in hospitalized patients without *C*. *difficile*. Also, our mouse data suggest that increased endogenous IL-33 is more likely a physiologic response to high-risk infection, given the protective role of exogenous IL-33 in stimulating protective humoral immunity.

We have shown an additional role for IL-33 signaling in mitigating dysbiosis by downregulating TH17 and TH1 cells to create an environment inadequate for *C*. *difficile* disease. TH17 cells are crucial in elevating the risk of severe CDI by serving as a significant source of IL17A (33). The sole adoptive transfer of TH17 cells has the potential to heighten the severity of CDI (33).

Understanding the role of IL-33 signaling in countering rCDI through the ILC2-TFH axis is crucial for crafting potent CDI vaccines. A significant hurdle is addressing antigenic variation, notably in TcdB, demanding a nuanced approach for broad-spectrum defense. A successful vaccine must navigate these complexities to ensure comprehensive protection. IL-33–associated mechanisms present a promising avenue for advancing CDI vaccine strategies, fostering optimism for more effective preventive measures. A proposition arises to enhance vaccine effectiveness by supplementing the antigen with a type 2–skewed adjuvant, amplifying IL-33 signaling and fortifying protection against CDI recurrence.

In summary, our investigation reveals that IL-33 prompts the expansion of ILC2s, which, in turn, either directly or indirectly amplify TFH cells, supporting B cells in antibody production. The resulting toxin-specific antibodies are essential in mitigating clinical illness due to reinfection**.**

## Methods

### Sex as a biological variable.

Our study examined male and female animals, and similar findings were reported for both sexes.

### Mice.

All animal procedures were approved by the Institutional Animal Care and Use Committee at the University of Virginia. C57BL/6J and μMT mice were purchased from the Jackson Laboratory, ST2^–/–^ mice were obtained from Dr. Andrew McKenzie (Laboratory of Molecular Biology, Cambridge University, Cambridge, UK), ROSA26^LSL–DTR^ (ROSA26iDTR; Jax 007900), transgenic Nmur1iCre-eGFP reporter mouse was gifted by David Artis group (26, 34). Sex-matched 8-to-12 week-old male or female mice were used in experiments. Animals were housed in a specific pathogen-free environment at the University of Virginia’s animal facility. The bedding was exchanged every 2 days for a minimum of 3 weeks to equilibrate their microbiota. Mice were infected with *C*. *difficile* as previously described (14). In short, for 3 consecutive days, mice received an antibiotic cocktail in drinking water consisting of 215 mg/L metronidazole (Hospira), 35 mg/L colistin (Sigma-Aldrich), 45 mg/L vancomycin (Mylan), and 35 mg/L gentamicin (Sigma-Aldrich), starting 6 days before infection. For the 3 days leading up to infection, regular drinking water was provided to the mice. A day before infection, clindamycin 0.016 mg/g (Hospira) was administered i.p. After infection, mice were monitored twice daily to evaluate clinical scoring parameters and weight loss over the course of infection and reinfection. The scoring criteria included weight loss, coat condition, eye condition, activity level, diarrhea, and posture (14). If mice reached a clinical score of 14 or lost more than 25% of weight, they were humanely euthanized. We defined the acute phase as when the mice had active diarrhea and lost weight. We defined recovery as when the mice started regaining weight.

### Bacterial strains and culture.

For the first infection, mice were infected with 5 × 10^4^ CFU/mL vegetative cells of either R20291 or VPI 10643 (ATCC 43244) strain. For reinfection, 1 × 10^6^ spores/mL of the R20291 strain or 5.2 × 10^4^ spores/mL of the VPI 10643 strain were used. *C*. *difficile* strains were plated on BHI agar from glycerol stocks and incubated overnight at 37° C in an anaerobic chamber (35). Columbia, clospore, and BHI broth were reduced for at least 24 hours.

### To generate spore stocks.

A single colony was inoculated into 15 mL of Columbia broth overnight at 37 °C, and then 5 mL of this culture was added to 45 mL of Clospore broth anaerobically and left for 7 days at 37°C (36). The culture was washed with cold sterile water at least 5 times and resuspended in 1 mL of sterile water. The spores were stored in a 1.5 mL twist cap tube at 4 °C (Corning 4309309).

### For vegetative infection.

A single colony was inoculated in BHI medium overnight at 37 °C. The next day, cultures were washed twice with anaerobic PBS. The concentrations were measured by optical density for R20291 infection. For infection with the VPI 10643 (ATCC 43244) strain, the overnight culture was subcultured for 5 hours before optical density measurement. The needed concentrations of vegetative cells were prepared and loaded into a syringe with a gavage needle inside the anaerobic chamber. Each mouse received 100 μL (5 × 10^3^ CFU for R20291 and 5 × 10^3^ CFU for VPI 10643) of inoculum by oral gavage. The actual inoculum was further verified by plating on BHI agar supplemented with 0.032 mg/mL cefoxitin, 1 mg/mL D-cycloserine, and 1% sodium taurocholate (Sigma-Aldrich), and incubating anaerobically at 37 °C overnight. *C*. *difficile* burden was measured either by toxin A– and toxin B–specific qPCR on the DNA isolated from the stool or cecal content using a QIAamp Fast DNA Stool Mini Kit according to the manufacturer’s instructions or by using an ELISA kit (TechLab Inc., TL5025) according to the manufacturer’s instructions to quantify bacterial count, normalized to stool or cecal content weight. The TOX A/B II ELISA kit (TechLab Inc., T5015) was used per the manufacturer’s instructions to quantify Toxin A/B, normalized to stool or cecal content weight.

### Antibodies.

HRP-conjugated anti-mouse IgM (1021-05), IgG (1031-05), IgG1 (1071-05), and IgA (1040-05) were purchased from Southern Biotech, and goat anti-human IgG and Fcg fragment specific antibodies from the Jackson Laboratory (109035-098).

### Tissue transcript and protein analysis.

Tissue lysates were obtained by washing the ceca with 1× PBS and then homogenizing them for 1 minute in 300 μL of lysis buffer I, which contained 5 mM HEPES and 1× HALT protease inhibitor (Pierce). The tubes were then incubated on ice for 30 minutes after adding 300 μL of buffer II, containing 5 mM HEPES, 1× HALT protease inhibitor, and 2% Triton X-100. The supernatant was collected by centrifuging at 13,000*g* at 4°C, and the total protein concentration was measured using a BCA assay, following the manufacturer’s instructions (Pierce). The mouse Duoset sandwich ELISA kit (R&D) was used to detect the IL-33 in the cecal tissue lysates, according to the manufacturer’s instructions.

For IL-33 mRNA transcript analysis, the RNeasy Mini Kit from Qiagen and DNase digestion (TURBO DNA-free Kit, Invitrogen) were used according to the manufacturer’s instructions. RNA from cecal tissue was stored in RNAlater at –80°C. Tetro cDNA Synthesis Kit (Bioline) was used to prepare the cDNA, and amplification of IL-33 was accomplished by the Taqman IL-33 Primer/Probe Set (Applied Biosciences, Mouse Assay ID: Mm00505403_m1). Normalization of gene expression was completed using HPRT and GAPDH housekeeping genes.

### Flow cytometry.

The colon was rinsed in a buffer containing HBSS, 25 mM HEPES, and 5% FBS. Dissociation buffer (HBSS with 15 mM HEPES, 5 mM EDTA, 10% FBS, and 1 mM DTT) was used to remove epithelial cells from the isolated colon for 40 minutes at 37 °C with 122 rpm agitation. Digestion buffer (RPMI 1640 containing 0.17 mg/mL Liberase TL [Roche] and 30 μg/mL DNase [Sigma-Aldrich]) was used to digest manually diced lamina propria for 40 minutes at 37 °C with 122 rpm agitation. After digestion, a 100 μM cell strainer followed by a 40 μM cell strainer (Thermo Fisher Scientific) was used to obtain single-cell suspensions. Extracellular staining was done with BB515-CD19 (BD 564509, dilution 1/25), PerCP-Cy5.5-CD5 (Biolegend, 100624, dilution 1/100), PerCP-Cy5.5-CD3 (Biolegend, 100218, dilution 1/100), PerCP-Cy5.5-FcεRIα (Biolegend, 134320, dilution 1/100), BV510-CD90 (Biolegend, 140319, dilution 1/25), BUV805-CD11b (eBiosciences/Thermo Fisher Scientific, 368-011282, dilution 1/400), PE-CY5-CD64 (Biolegend, 139332, dilution 1/75), APC-Fire 810-Ly6C (Biolegend, 128055, dilution 1/400), BV785-CD45 (Biolegend, 103149, dilution 1/200), NovaFluor Blue 610-70s-CD8a (Thermo Fisher Scientific, M003T02B06, dilution 1/200), AF700-CD4 (Biolegend, 100430, dilution 1/400), FITC-TCRbeta (Biolegend, 159706, dilution 1/50), BV605-TCRgd (Biolegend, 118129, dilution 1/200), BV650-CD11c (Biolegend, 117339, dilution 1/100), APC Cy7-CD103 (Biolegend, 121432, dilution 1/30), Pacific blue-CD40 (Biolegend, 124626, dilution 1/50), AF647-CD80 (Biolegend, 104718, dilution 1/100), PE-CD86 (Biolegend, 105008, dilution 1/50), BUV 737-Ly6G (Thermo Fisher Scientific, 367-9668-82, dilution 1.25/100), Spark UV 387-MHCII (Biolegend, 107670, dilution 1/400), BUV 563-SiglecF (Thermo Fisher Scientific, 365-1702-82, dilution 1/400), PE-Dazzle 594-CD127 (Biolegend, 135032, dilution 1/100), PE Dazzle 594-CXCR5 (Biolegend, 145522, dilution 1/100), FITC-CD44 (Biolegend, 103005, dilution 1/100), BV510-PD1 (Biolegend, 135241, dilution 1/100). Intracellular staining was done with BV421-Tbet (Fisher Scientific/BD Biosciences, 5563318, dilution 1/20), APC-RorgT (Thermo Fisher Scientific, 1769818, dilution 1/33), BV711-GATA3 (Fisher Scientific/BD Biosciences, 565449, dilution 1/50), PerCP-eFluor 710-FOXP3 (Thermo Fisher Scientific 46577382, dilution 1/100), and PE-fire 700-CD206 (Biolegend, 141741, dilution 1/75). For surface staining, 1 × 10^6^ cells/sample were Fc-blocked with TruStain fcX (BioLegend, 101320, dilution 1/200) for 10 minutes at room temperature followed by the addition of LIVE/DEAD blue (Thermo Fisher Scientific L34962) for 30 minutes at 4°C. Cells were washed twice in FACS buffer (PBS + 2% FBS) and stained with fluorochrome-conjugated antibodies for 30 minutes at 4°C. Cells were washed and resuspended in Foxp3 Fix/Perm WorkinFg Solution (ebiosciences, 00-5523-00) and incubated overnight at 4°C. Cells were washed twice with permeabilization buffer and stained for 30 minutes at room temperature. Flow cytometry was performed on a Cytek Aurora (5-Laser) Spectral Flow Cytometer and analysis was done on Omiq software. All cell counts were normalized based on 80,000 live cell counts. SpectroFlo QC beads (SKU B7-10001) were used for routine performance tracking of the Cytek Aurora (5-Laser) Spectral Flow Cytometer. Unmixing was performed using single stains prepared on either cells or UltraComp eBeads Plus Compensation Beads (01-3333-42). All the gating strategies used in the article are presented in Supplemental Figure 10.

### IL-33 and anti-CD20 treatment.

Carrier-free recombinant mouse IL-33 (Biolegend; 580504) was diluted with sterile PBS to prepare a 7.5 μg/ml solution. 100 μL was injected intraperitoneally daily for 5 days prior to the first infection or reinfection. Anti-CD20 was a gift from Genentech. A total of 250 μg of anti-CD20 per mouse was injected intraperitoneally on days –7, 3, 18, 33, 48, and 63.

### ELISA.

96-well half-area assay plates (Corning) were used to detect toxin-B-specific antibodies. Plates were coated with 2 μg/mL of toxin B in carbonate buffer (Sigma-Aldrich), a generous gift from Techlab, and kept at 4°C overnight. The next day, plates were blocked with 1% BSA in PBS and 0.05% Tween 20 at 37°C for 1 hour. Samples were added by diluting mouse sera, fecal supernatant, or cecal content in PBS-T, and plates were incubated for another 1 hour at 37°C. *C*. *difficile* toxin B monoclonal antibody (clone A13I, catalog MA17412; Thermo Fisher Scientific), was used as a positive control. HRP-conjugated IgG (1:5,000), IgG1 (1:5,000), IgM (1:1,000), or IgA (1:1,000) was added after washing 3 times with PBS-T. Wells were developed by either 2,2′-azinobis (3-ethylbenzthiazolinesulfonic acid) (ABTS) substrate (KPL) or Ultra TMB-ELISA substrate solution (Thermo Fisher Scientific) at room temperature for 10 minutes and stopped by either 1% SDS or 2 M H_2_SO_4_. Optical density for ABTS substrate was read at 410 nm with a background at 650 nm. The optical density for the TMB substrate was read at 450 nm with a background at 630 nm. A well containing control plasma was used as the negative control.

### ILC2 adoptive transfer studies.

MLN, spleen, colon, and cecum were extracted from WT C57BL/6J mice that had received 5 daily doses of IL-33 (0.75 μg). A single-cell suspension was prepared from the colon and cecum, as described above. For MLN and spleen, a 40 μM cell strainer (Thermo Fisher Scientific) was used to prepare a single-cell suspension. After passing through the 40 μM cell strainer, 1 mL of 1 × RBC lysis buffer (Thermo Fisher Scientific) was added for 1 minute. After centrifugation at 600*g* for 6 minutes, single cells were prepared in FACS buffer containing 2% heat-inactivated FBS in PBS. The single cells obtained from MLN, spleen, cecal, and colon were subjected to lineage-positive cell depletion by magnetic bead purification of lineage-negative (Lin^–^) populations (bulk ILCs) (Miltenyi Lineage Cell Depletion Kit: 130-110-470). The lin^–^ cells were expanded in vitro in complete RPMI 1640 media containing 10% FBS, 2 mM glutamine, 100 U/mL penicillin, 100 μg/ml streptomycin, 50 ng/mL of IL-33, and 10 ng/mL of IL-2 and IL-7 for 4 days. Cells were flow sorted on the Influx Cell Sorter (BD Biosciences) based on Lin^–^ (CD11c, CD3, CD5, CD11b, CD19, Fc epsilon R1 α with PECy7 fluorochrome) CD45^+^ CD90.1^+^ CD127^+^ CD25^+^ ST2^+^ expression after cell surface staining. Approximately 3 × 10^5^ ILC2s were transferred into each ST2^–/–^ mouse.

### Detection of Human IL-33 in serum.

Male and female hospitalized patients between 18 and 90 years of age with diarrhea and a positive CDI PCR test (nucleic acid amplification test [NAAT] GeneXpertSerum) were approached to be in the study. Patient demographics and clinical data were collected, and a follow up was completed on all participants to determine CDI recurrence and mortality after enrollment. Consented and enrolled participants had 20 mL of blood drawn in EDTA tubes within 24 hours of CDI diagnosis. The blood was spun down at 2000*g* for 15 minutes at room temperature and plasma was stored in aliquots at –80°C until use, at which point they were thawed on ice. Plasma was collected from 17 healthy donors without *C*. *difficile* infection, and 58 prospectively enrolled hospitalized patients within 48 hours of diagnosing *C*. *difficile* infection. Among *C*. *difficile*–infected patients, clinical outcomes (recurrent infection or death) were measured over an 8-week follow-up period. Recurrent *C*. *difficile* infection was defined as symptom relapse following completion of treatment for the index episode, requiring retreatment. Serum cytokine concentrations were measured using a commercial multiplex proximity extension assay (Olink Proteomics). The lower limit of detection for our assay is 0.24 pg/mL. Any values below this threshold were reported as zero, which may influence our data interpretation. Concentrations of IL-33 were compared between healthy controls, patients who survived the follow-up period without recurrent *C*. *difficile* infection, and those who developed a complication (recurrence or death).

### Mouse and human histology and IHC.

Proximal colonic sections were fixed in Bouin’s solution and transferred to 70% ethanol after 24 hours. Staining was done with either H&E or Periodic Acid Schiff (PAS) after preparing paraffin-embedded sections by the University of Virginia Research Histology Core. Two blinded observers scored histopathology using a scale from 0 to 3 for submucosal edema: 0, none; 1, mild; 2, moderate; and 3, intense/severe damage. Epithelial disruption and immune cell infiltration were scored using the same scale. Hemorrhage was scored as 1, yes; 0, no (37). Goblet cells were identified as PAS^+^ and their number normalized to the number of crypts. Human biopsies sourced from the University of Virginia Biorepository and Tissue Research Facility were utilized. Researchers were kept blind to patient identities. The University of Virginia Biorepository Core conducted staining of human colon biopsy sections using a primary antibody targeting IL-33 (R&D, AF3625, diluted at 1/80,000).

### Flow cytometry of human PBMCs.

PBMCs were isolated from whole blood by centrifugation to remove plasma, followed by Ficoll (Cytiva) density gradient centrifugation in SepMate tubes (Stemcell Technologies). The isolated PBMCs were counted, and 2 million cells were cryopreserved in liquid nitrogen until staining. Antibodies used for staining are listed in Table 1. Controls included unstained, single-stained, and fluorescence-minus-1–stained (FMO-stained) PBMCs. A fixed viability stain (Live/Dead Blue; Thermo Scientific L23105) was applied to each sample. Samples were analyzed on a 5-laser Cytek Aurora Borealis flow cytometer, with 130,000 cells collected per sample. Fluorescence data were analyzed using OMIQ to phenotype T cell subsets through traditional gating (Supplemental Figure 12C). Spectral deconvolution and gating were based on single-stained and FMO-stained PBMC control samples.

### FITC-Dextran gut permeability assay.

To assess intestinal permeability, mice were orally administered a FITC-dextran solution (Sigma-Aldrich, 46944-500MG-F) at a dosage of 44 mg per 100 g body weight. 4 hours after administration, mice were euthanized, and serum samples were collected. The concentration of FITC-dextran in the serum was measured using a spectrophotometer set at excitation and emission wavelengths of 485 nm and 530 nm, respectively.

### Statistics.

The Kaplan–Meier method was used to measure recurrence-free survival curves and to evaluate the effects of IL-33 on risk for recurrent *C*. *difficile* infection. To account for competing risk against recurrent infection, death within the 8-week follow-up period was treated as a censoring event. A 2-tailed *t* test for normally distributed data, a Mann-Whitney test for nonnormally distributed data (serum IL-33), Šídák’s for multiple comparisons were used to determine the statistical significance between groups. *P* = 0.05 is considered statistically significant. Statistical analyses were performed using GraphPad Prism software (GraphPad Software Inc. or R version 4.2.0 (R Core Team).

### Study approval.

The collection and analyses of patient samples and healthy controls were approved by the University of Virginia Institutional Review Board (IBR-HSR18782 and HSR220013) . Participants provided written informed consent prior to participation in the study. All animal procedures were approved by the Institutional Animal Care and Use Committee at the University of Virginia (IACUC).

### Data availability.

Supporting data values for all the generated data are available in the Supporting Data Values file for reference. Further information and requests for reagents will be fulfilled by the corresponding author WP or FN (Ymw4xw@virginia.edu).

## Author contributions

FN and WAP designed all of the experiments. FN performed the experiments, analyzed and interpreted data, and wrote the manuscript. JU and NH helped with tissue extraction and processing. RB and ACB helped with mouse tissue extraction. DT helped in tissue biopsies scoring. MKY, GR, IR, and GRM helped in human studies. CF helped in ILC2s knock-out mouse breeding. GRM, GR, DT, and WAP edited the manuscript. WAP supported all aspects of the work.

## Supplementary Material

Supplemental data

Supporting data values

## Figures and Tables

**Table 1:**
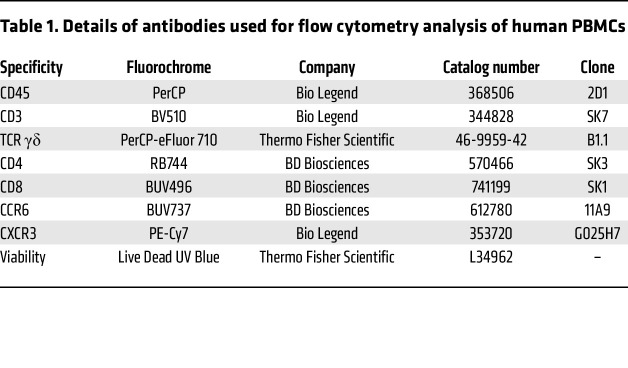
Details of antibodies used for flow cytometry analysis of human PBMCs

## Version 1 03/06/2025

In-Press Preview

## Version 2 05/01/2025

Electronic publication
