# Supplementary material for: IL-33 protects from recurrent C. difficile infection by restoration of humoral immunity
Source: J Clin Invest. 2025 Mar 6;135(9):e184659. doi: 10.1172/JCI184659 (PMC12043089; doi:10.1172/JCI184659)
Supplement: Supplemental data [file jci-135-184659-s186.pdf]

| | **Uncomplicated CDI (n=39)** | **Recurrent CDI within 90 days (n=12)** | **Death within 90 days (n=5)** | **P value** |
|---|---|---|---|---|
| Female Sex | 20 (51.3%) | 7 (58.3%) | 4 (80%) | 0.14 |
| Age in a year, Mean (IQR) | 56.5 (46-67) | 59.3 (52-71.5) | 72.4 (63-80.5) | **0.02** |
| Race/ethnicity: | | | | 0.07 |
| White/Caucasian | 30 (76.9%) | 10 (83.3%) | 3 (60%) | **0.05** |
| Black/African-American | 7 (17.9%) | 2 (16.7%) | 2 (40%) | **0.03** |
| Hispanic/Latino | 1 (2.6%) | 0 (0%) | 0 (0%) | 0.51 |
| Asian/Other | 1 (2.6%) | 0 (0%) | 0 (0%) | 0.51 |
| Peak WBC, Mean (IQR) | 12.6 (6.1-16.3) | 12.2 (3.0-19.2) | 14.1 (6.2-22.6) | 0.82 |
| Charlson Comorbidity Index, Mean (IQR) | 4.3 (2-6) | 4.8 (2.5-7) | 8 (6-10) | **0.001** |

**Supplemental Table 1: Basic patient characteristics.**

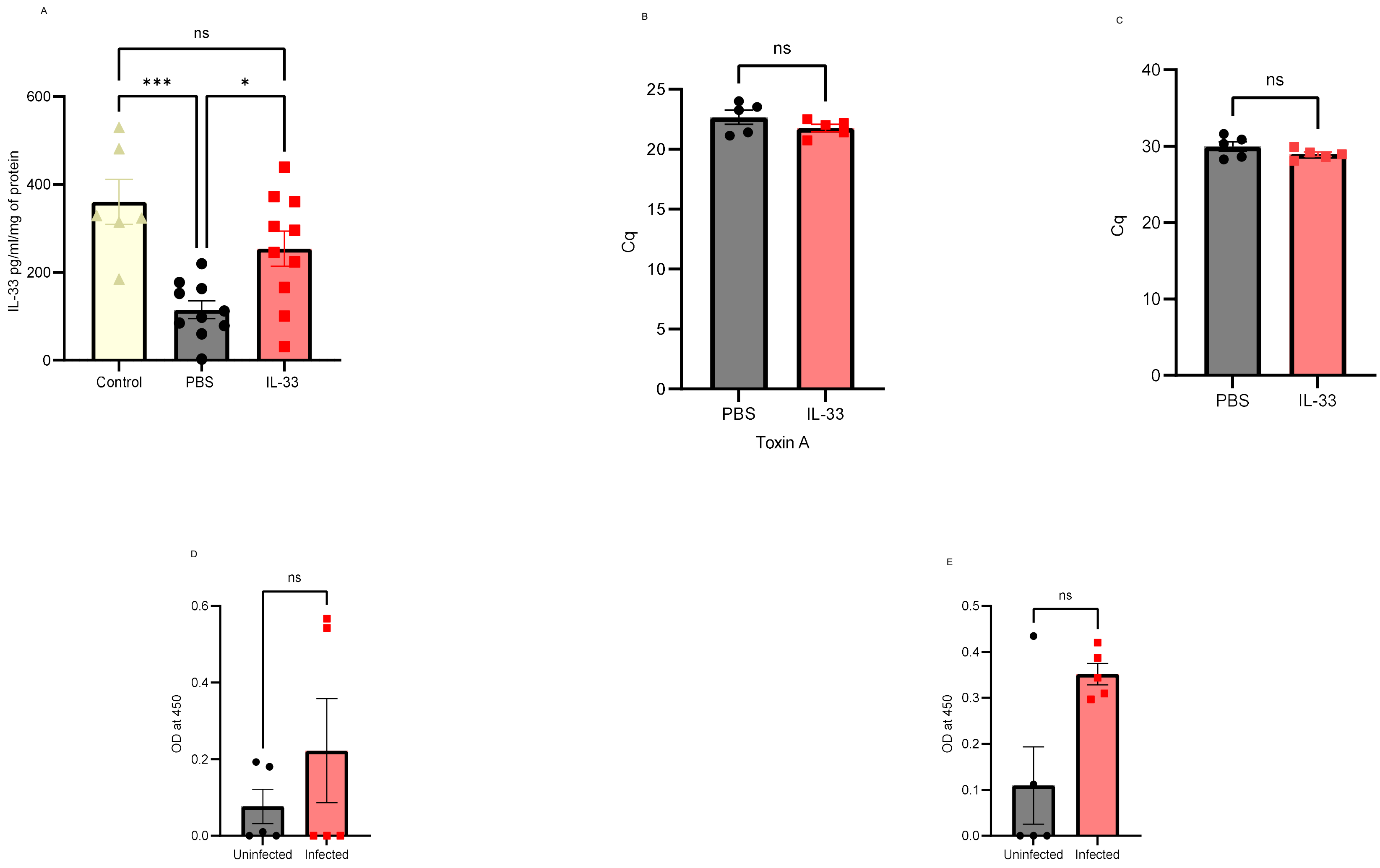


Supplemental Figure 1.

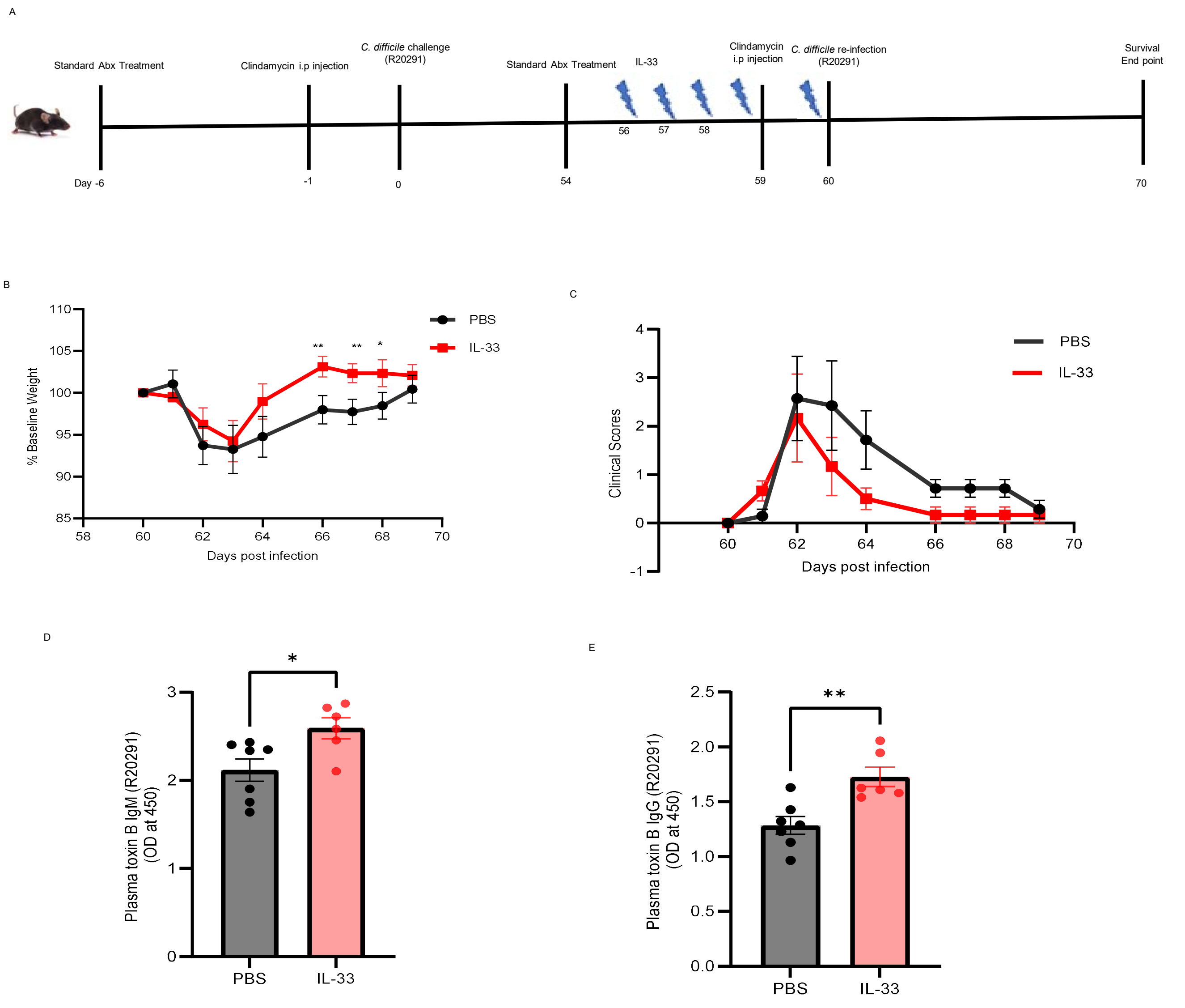


Supplemental Figure 2.

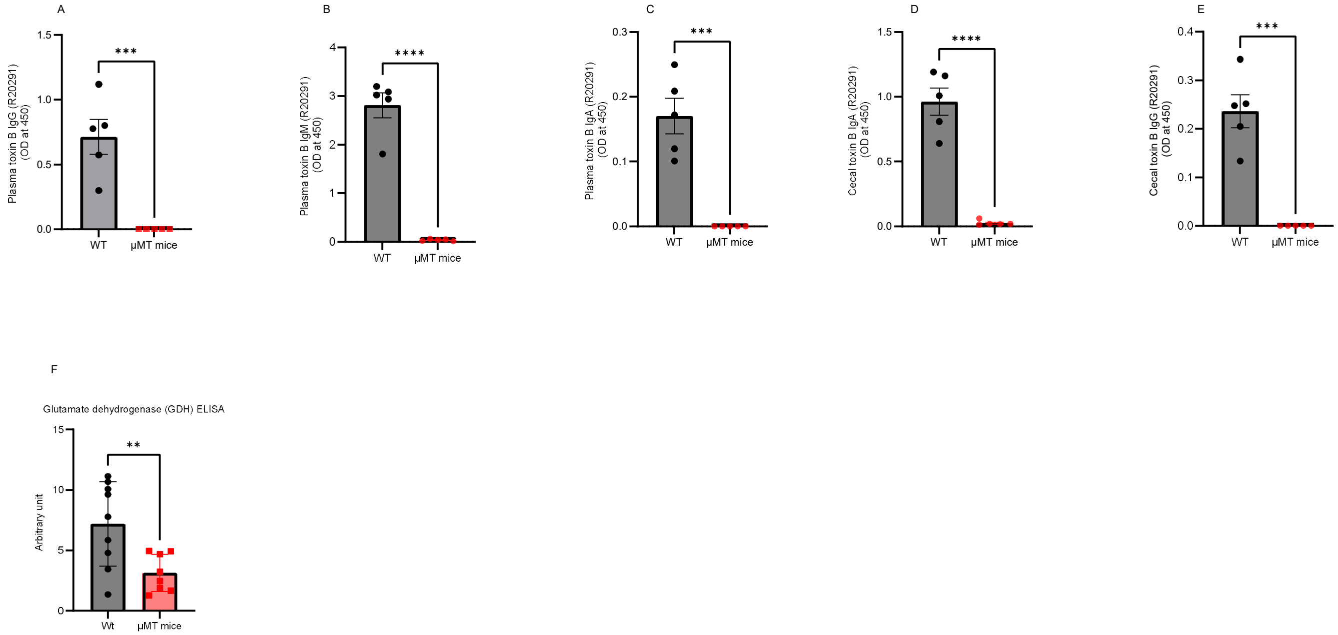


Supplemental Figure 3.

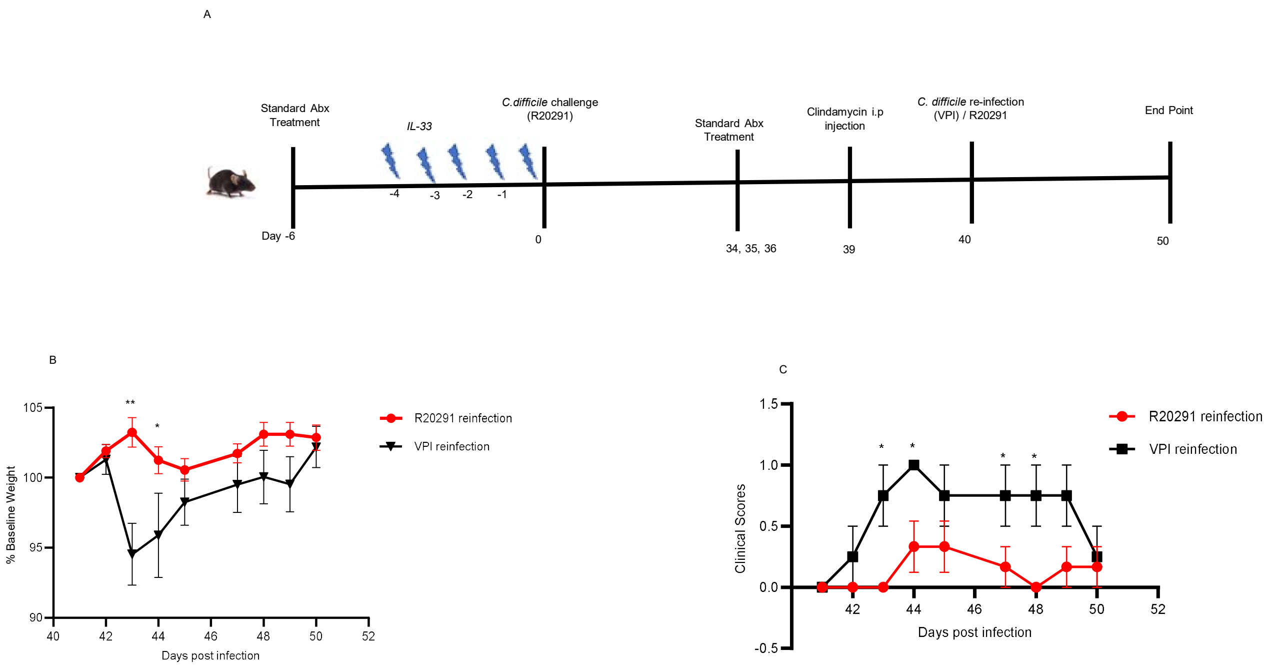


Supplemental Figure 4.

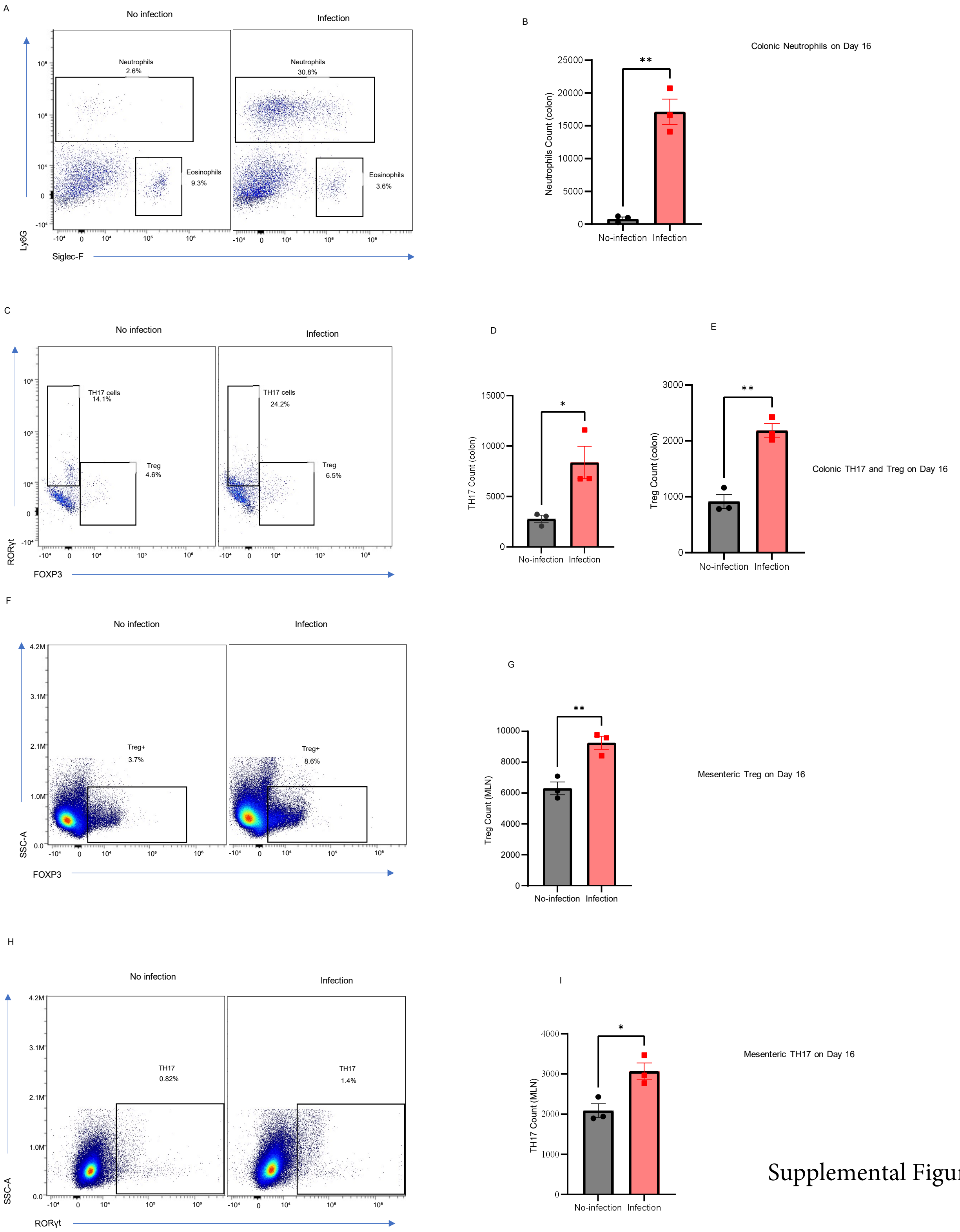


Supplemental Figure 5.

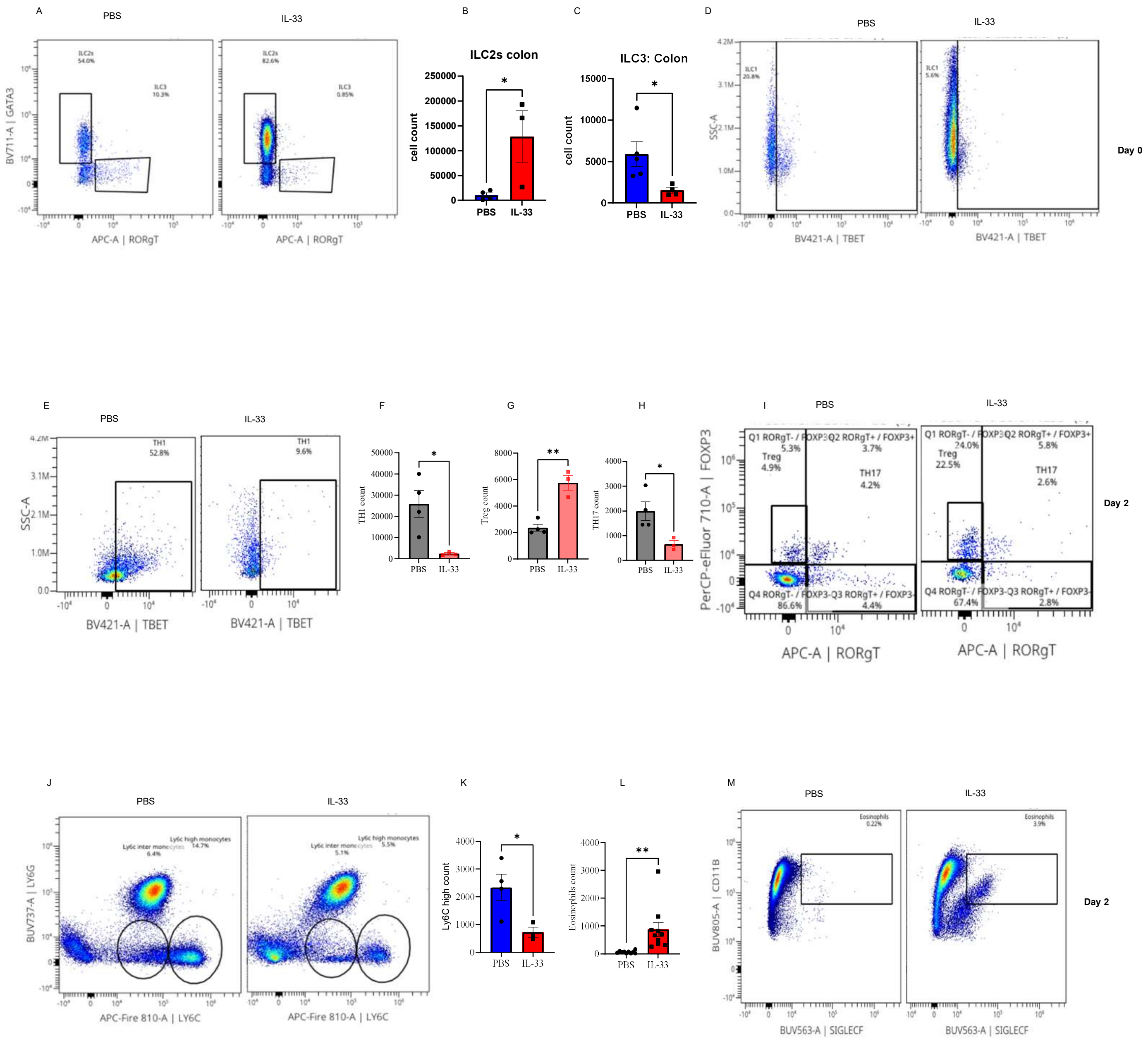


Supplemental Figure 6.

A

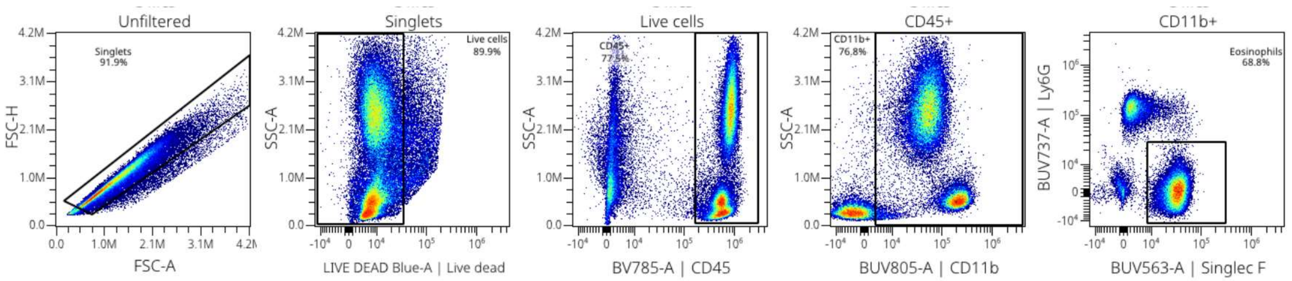


B

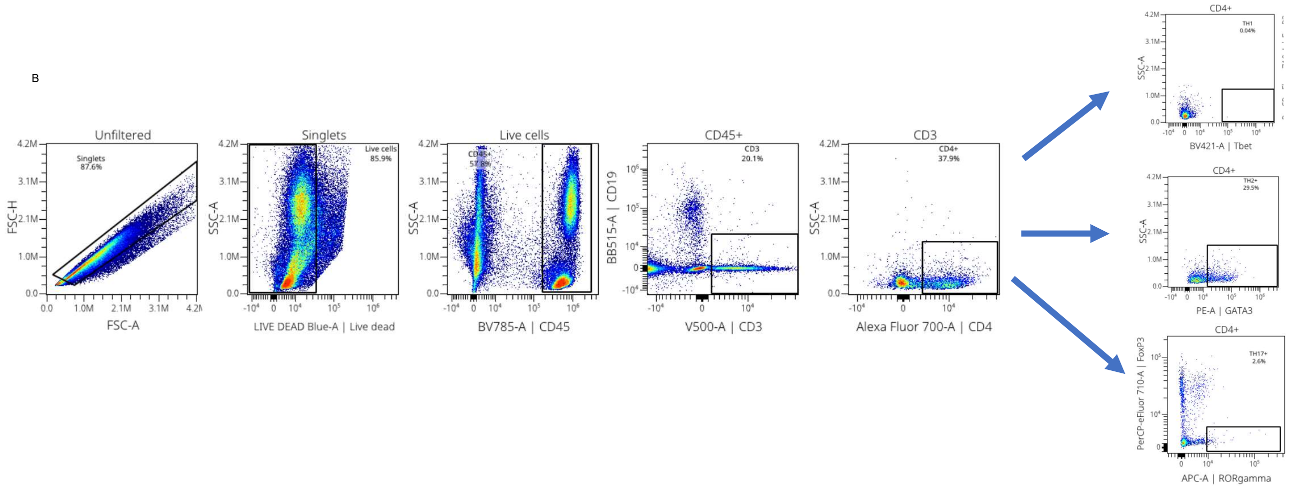


Supplemental Figure 7.

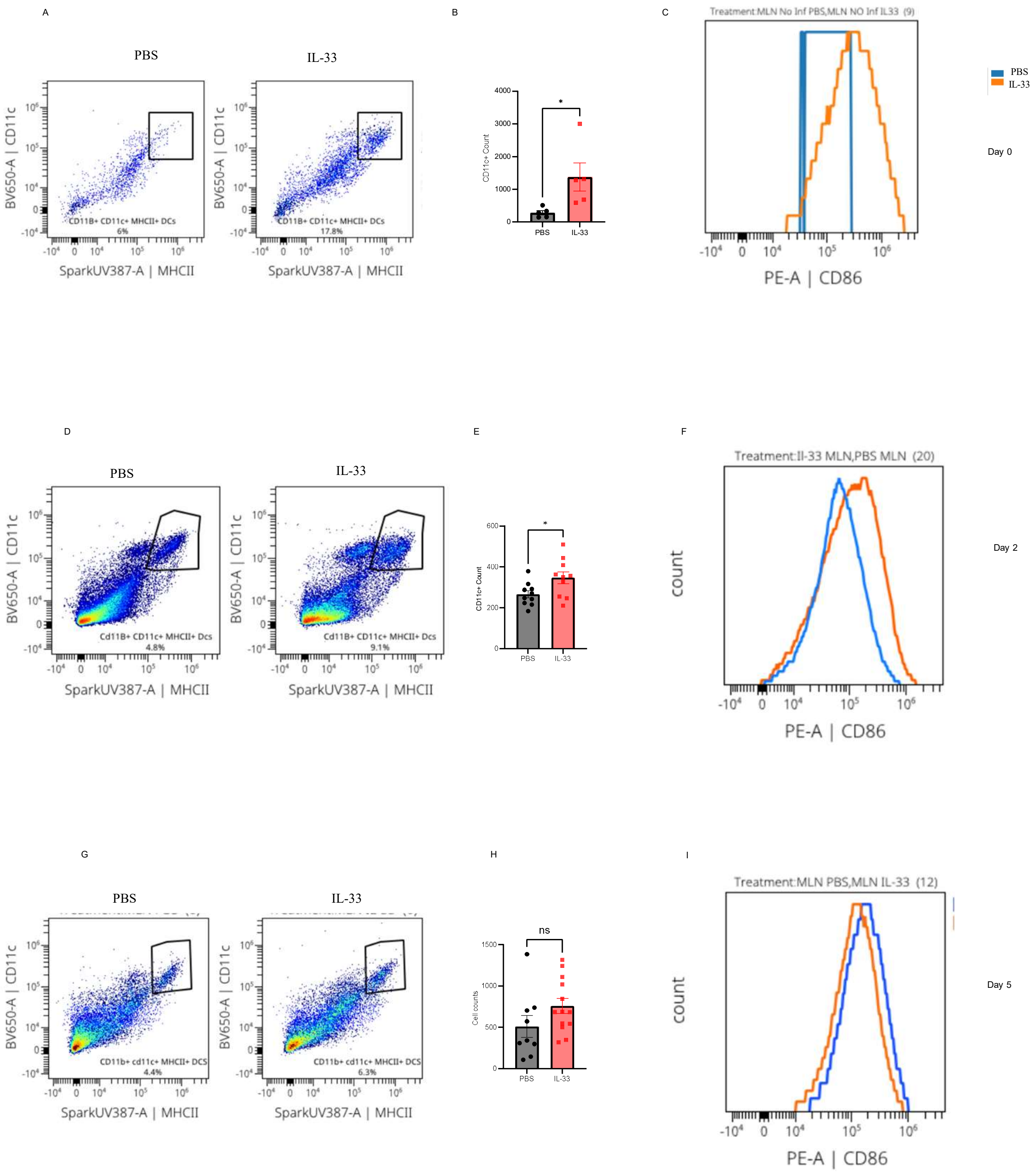


Supplemental Figure 8.

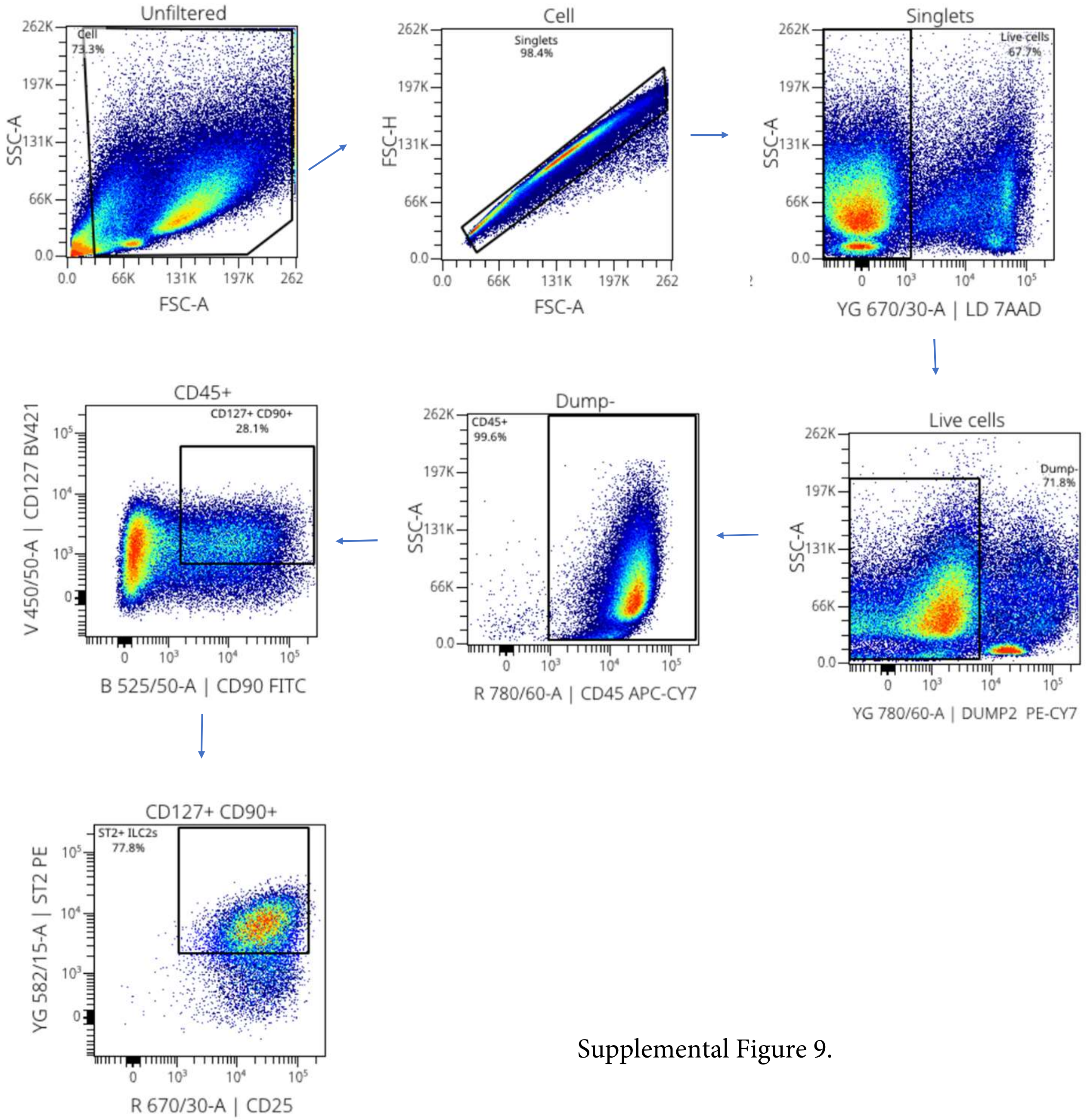


Supplemental Figure 9.

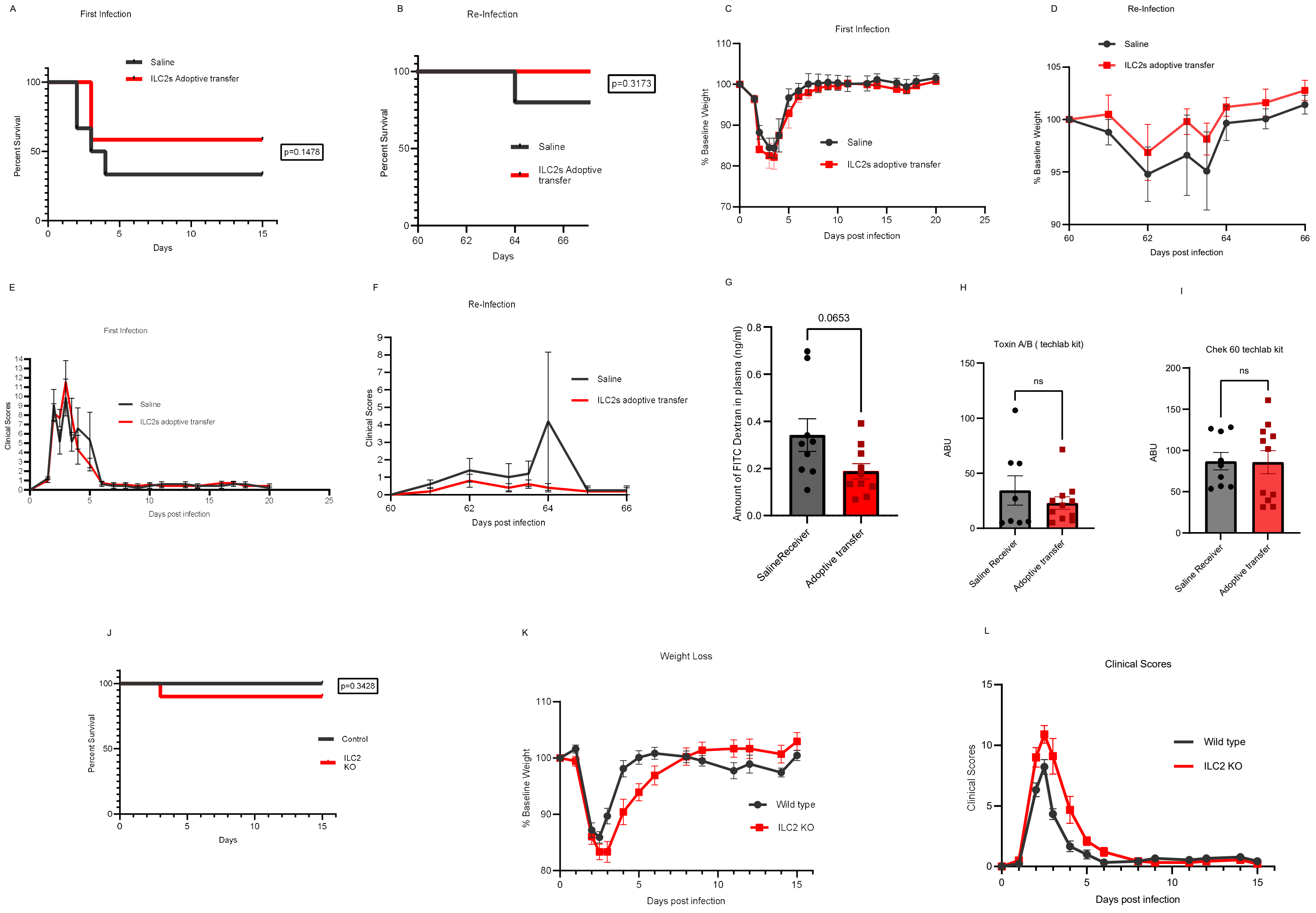


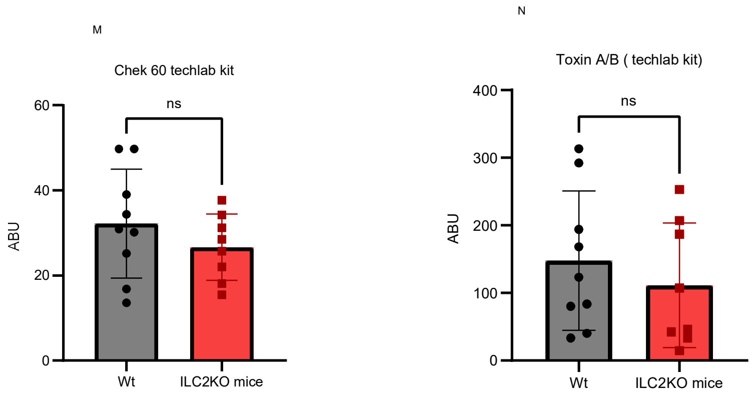


Supplemental Figure 10.

A

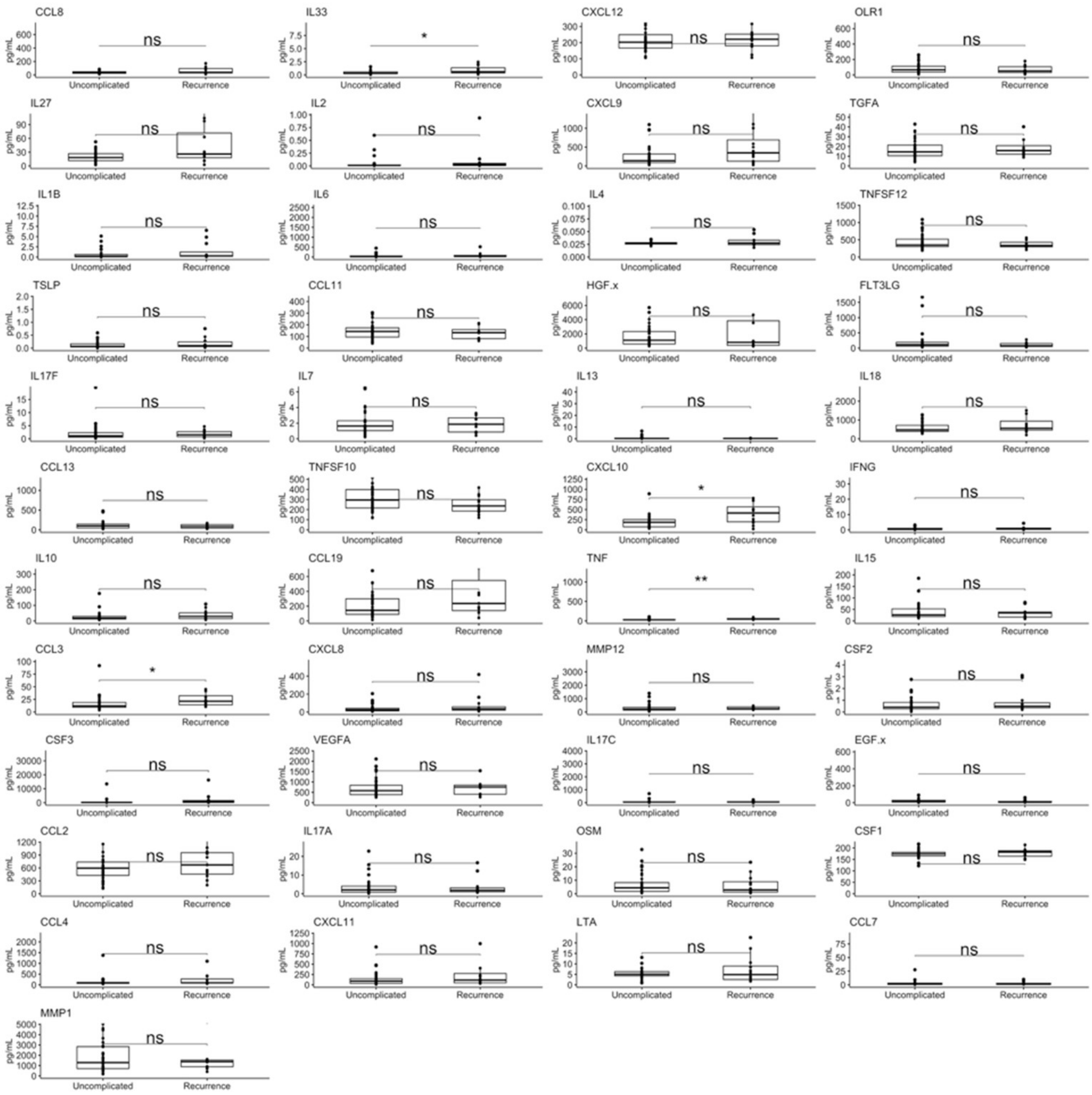


B

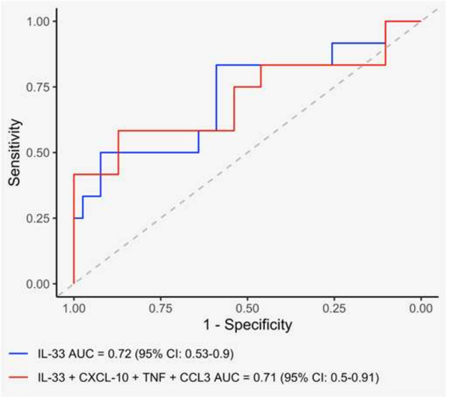


Supplemental Figure 11.

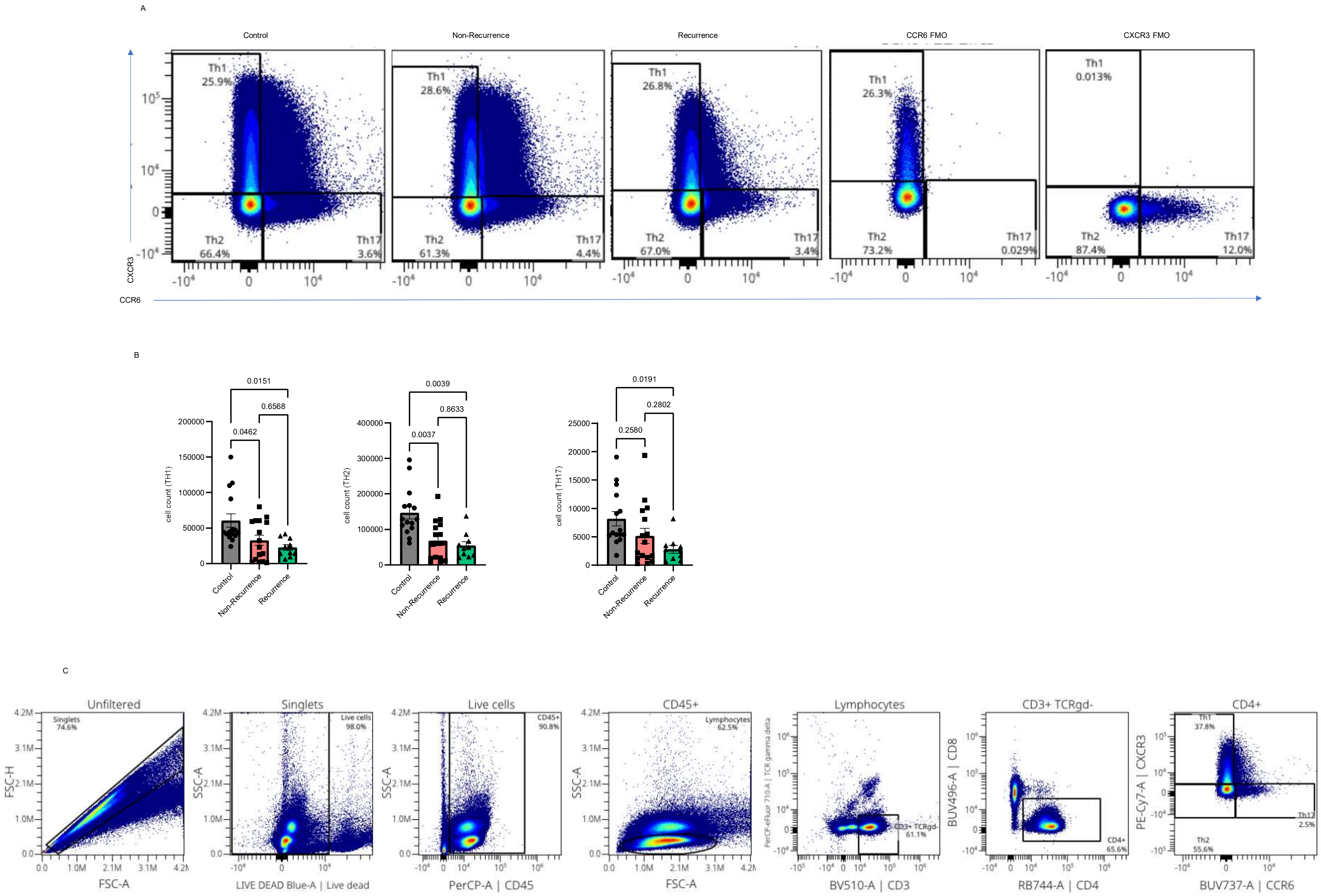


Supplemental Figure 12.
